# Delineating selective vulnerability of inhibitory interneurons in Alpers' syndrome

**DOI:** 10.1111/nan.12833

**Published:** 2022-07-19

**Authors:** Laura A. Smith, Daniel Erskine, Alasdair Blain, Robert W. Taylor, Robert McFarland, Nichola Z. Lax

**Affiliations:** ^1^ Wellcome Centre for Mitochondrial Research, Faculty of Medical Sciences Newcastle University Newcastle upon Tyne UK; ^2^ Translational and Clinical Research Institute, Faculty of Medical Sciences Newcastle University Newcastle upon Tyne UK; ^3^ NHS Highly Specialised Service for Rare Mitochondrial Disorders of Adults and Children Newcastle upon Tyne Hospitals NHS Foundation Trust Newcastle upon Tyne UK

**Keywords:** Alpers' syndrome, calretinin, inhibitory interneurons, mitochondrial epilepsy, parvalbumin, POLG, seizures

## Abstract

**Aims:**

Alpers' syndrome is a severe neurodegenerative disease typically caused by bi‐allelic variants in the mitochondrial DNA (mtDNA) polymerase gene, *POLG*, leading to mtDNA depletion. Intractable epilepsy, often with an occipital focus, and extensive neurodegeneration are prominent features of Alpers' syndrome. Mitochondrial oxidative phosphorylation (OXPHOS) is severely impaired with mtDNA depletion and is likely to be a major contributor to the epilepsy and neurodegeneration in Alpers' syndrome. We hypothesised that parvalbumin‐positive(+) interneurons, a neuronal class critical for inhibitory regulation of physiological cortical rhythms, would be particularly vulnerable in Alpers' syndrome due to the excessive energy demands necessary to sustain their fast‐spiking activity.

**Methods:**

We performed a quantitative neuropathological investigation of inhibitory interneuron subtypes (parvalbumin+, calretinin+, calbindin+, somatostatin interneurons+) in postmortem neocortex from 14 Alpers' syndrome patients, five sudden unexpected death in epilepsy (SUDEP) patients (to control for effects of epilepsy) and nine controls.

**Results:**

We identified a severe loss of parvalbumin+ interneurons and clear evidence of OXPHOS impairment in those that remained. Comparison of regional abundance of interneuron subtypes in control tissues demonstrated enrichment of parvalbumin+ interneurons in the occipital cortex, while other subtypes did not exhibit such topographic specificity.

**Conclusions:**

These findings suggest that the vulnerability of parvalbumin+ interneurons to OXPHOS deficits coupled with the high abundance of parvalbumin+ interneurons in the occipital cortex is a key factor in the aetiology of the occipital‐predominant epilepsy that characterises Alpers' syndrome. These findings provide novel insights into Alpers' syndrome neuropathology, with important implications for the development of preclinical models and disease‐modifying therapeutics.

## Graphical abstract

Alpers' syndrome is a severe mitochondrial disease characterised by intractable epilepsy and extensive neurodegeneration. Our neuropathological study has revealed an increased vulnerability of parvalbumin‐positive inhibitory interneurons to dysfunction and degeneration in Alpers' syndrome which likely underlies the severe occipital‐predominant epilepsy in patients with Alpers' syndrome. These findings have important implications for the development of preclinical models of Alpers' syndrome neuropathology and disease‐modifying therapeutics.

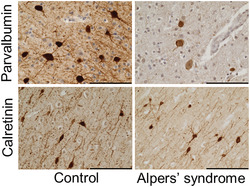

**Key points**

- Intractable epilepsy with occipital focus is a major presenting feature of Alpers' syndrome, but the aetiological factors underlying this focus in the primary visual cortex are not known.
- Parvalbumin‐positive(+) interneurons are typically fast‐spiking inhibitory interneurons which have high metabolic demands and are, thus, particularly vulnerable to mitochondrial dysfunction.
- We performed one of the largest *postmortem* studies of Alpers' syndrome to date, focusing on interneuron populations in occipital cortex compared with frontal and temporal, demonstrating degeneration of parvalbumin+interneurons and mitochondrial oxidative phosphorylation (OXPHOS) deficiencies in residual cells.
- Comparison of regional abundances of interneuron subtypes demonstrated markedly higher numbers of parvalbumin+ interneurons in control occipital cortex, likely indicating a reliance on this subtype for optimal functioning of occipital cortex under normal physiological conditions.
- The apparent vulnerability of parvalbumin+ interneurons, and the enrichment of parvalbumin+ interneurons in the primary visual cortex, may underlie the occipital‐predominant epilepsy in Alpers' syndrome.

## INTRODUCTION

Alpers' syndrome is a devastating mitochondrial disease characterised by intractable epilepsy, psychomotor regression and hepatic failure [1, 2, 3]. Focal seizures within the primary visual cortex are a typical early presenting feature and are often refractory to treatment [4]. Other seizure types including myoclonic, generalised tonic–clonic and focal motor seizures are also observed and may lead to status epilepticus or more frequently, *epilepsia partialis continua* [5]. Additional neurological impairments are common and include developmental delay, cerebellar ataxia, cortical blindness and hypotonia [6]. Symptoms often manifest during early infancy; however, a second peak of onset occurs in adolescence or early adult life [7, 8]. Neurological deterioration is rapidly progressive, with fatal consequences occurring within a few months to years of initial presentation.

Alpers' syndrome is predominantly caused by bi‐allelic pathogenic variants in *POLG*, which encodes the catalytic subunit of DNA polymerase gamma [5, 9, 10]. Pathogenic *POLG* variants result in inefficient replication of mitochondrial DNA (mtDNA) leading to mtDNA depletion, particularly affecting brain and liver [9, 11, 12].

MtDNA depletion results in decreased activities of mitochondrial oxidative phosphorylation (OXPHOS) complexes and impaired generation of adenosine triphosphate (ATP), which leads to severe neural dysfunction and neurodegeneration [12]. These neurodegenerative changes are most profound within the primary visual cortex with focal necrotic cortical lesions characterised by severe neuronal dropout and gliosis [6, 7]. Focal cortical lesions correlate with the clinical onset of stroke‐like episodes (SLE) and are frequently observed on cranial magnetic resonance imaging as T2‐weighted hyperintensities that are not confined to a single vascular territory [13]. Although the precise pathophysiology of these events remains unclear, they are hypothesised to result from prolonged focal epileptic activity and acute neuronal energy failure resulting in near‐total neuronal loss [12].

Recent neuropathological investigations have shown a profound loss of gamma aminobutyric acid (GABA)‐ergic inhibitory interneurons from the nonlesional primary visual cortex in Alpers' syndrome, accompanied by severe deficiencies of OXPHOS complexes I and IV within residual interneurons [14]. The severe dysfunction and degeneration of inhibitory interneurons likely underlies a loss of inhibitory neurotransmission that creates a permissive environment for the generation and perpetuation of seizure activity. Similar levels of interneuron loss and multiple OXPHOS deficiencies have been reported in other mitochondrial epilepsies including adult‐onset POLG‐related pathologies [15].

Different subtypes of inhibitory interneurons have distinct topographies and electrophysiological properties which mediate the differential roles of interneuron subtypes in regulating cortical networks and their involvement in epilepsy [16, 17]. Parvalbumin‐positive (+) interneurons, which are typically fast‐spiking basket cells and underlie gamma frequency oscillations, are enriched with mitochondria and harbour particularly high levels of cytochrome *c* compared with other interneuron subtypes [18, 19, 20]. The increased metabolic requirements of parvalbumin+ interneurons to sustain their fast‐spiking activity render them particularly vulnerable to OXPHOS defects and ATP depletion [21]. Incubation of rodent hippocampal slices with rotenone and potassium cyanide, inhibitors of complex I and complex IV respectively, abolishes gamma frequency oscillations in vitro [22]. This highlights the critical dependence of OXPHOS for optimal function of parvalbumin+ interneurons and implicates an increased vulnerability of these interneurons in Alpers' syndrome where OXPHOS function is severely impaired. The loss of inhibitory neurotransmission associated with impaired function of parvalbumin+ interneurons would alter physiological cortical rhythms and permit an unrestrained escalation of excitatory activity leading to epileptic seizures.

This study aimed to determine whether specific interneuron subtypes are differentially affected in Alpers' syndrome, with a particular focus on parvalbumin+ interneurons. This will provide a greater insight regarding the role of impaired inhibitory neurotransmission in the generation of epileptic activity and may explain the early and predominant involvement of the occipital cortex in Alpers' syndrome. Better understanding of these pathomechanisms and cell‐specific sensitivity to OXPHOS deficiency will aid the development of accurate, relevant, preclinical models of Alpers' syndrome and allow testing of novel antiepileptic therapies.

## MATERIALS AND METHODS

### Patient and control cohort

Formalin‐fixed, paraffin‐embedded human postmortem brain tissues were obtained from three brain regions: occipital cortex (Brodmann Area 17), the predominant site of epileptogenesis in Alpers' syndrome and the dorsolateral prefrontal cortex (Brodmann Area 9) and inferior temporal cortex (Brodmann Area 20) as comparison cortical regions with less involvement in Alpers' syndrome (supporting information Table S1). Eight patients with clinically defined Alpers' syndrome, three patients with genetically confirmed Alpers' syndrome and three young adult patients with genetically confirmed POLG‐related encephalopathy were included in the study and were compared with age‐matched, cognitively normal control tissues. All patients with mitochondrial disease were grouped together for analyses and are referred to as patients with Alpers' syndrome from hereafter. The control cohort included nine individuals with no neurological or neurodegenerative diagnoses and five individuals diagnosed with sudden unexpected death in epilepsy (SUDEP; epilepsy disease control). SUDEP cases were defined as a premature unexplained death of an epilepsy patient for which there are no causes of death identified at autopsy and were included to control for generalised seizure‐associated pathology [23]. The study obtained ethical approval for use of these postmortem tissues from the respective brain banks, and informed consent was acquired from all cases. Tissues were matched, as closely as possible, for age (Kruskal–Wallis, *P* = 0.145), sex (*X* ^2^ test for trend, *P* = 0.891), postmortem interval (Kruskal–Wallis, *P* = 0.117) and duration of formalin fixation (Kruskal–Wallis, *P* = 0.756).

### Immunohistochemistry for the identification of cortical neuronal subtypes

Immunohistochemistry to identify parvalbumin+, calretinin+, calbindin+ and somatostatin+ interneurons and pyramidal neurons was performed using 5 μm thick formalin‐fixed, paraffin‐embedded sections as previously described (supporting information Table S2) [24]. These interneuron subtypes were investigated due to the known involvement in temporal lobe epilepsy (TLE) and the vulnerability of parvalbumin+ interneurons to mitochondrial dysfunction [22, 25, 26, 27, 28, 29]. Immunohistochemistry to identify reactive astrocytes and activated neurons (c‐fos) was also performed to neuropathologically characterise cortical lesions.

### Neuronal density (ND) quantification

Neuronal densities (neuron/mm^2^) were quantified using a two‐dimensional neuronal cell counting protocol on an Olympus BX51 stereology brightfield microscope as previously described [14, 15]. Quantification was performed in nonlesioned cortex; however, this was unavoidable for Patients 4–6 and Patients 10–11 as all cortical tissue was severely necrotic. Using control ND data, *z*‐scores were calculated ([log‐transformed ND – mean control log‐transformed ND]/SD control log‐transformed ND) in order to make inferences about the severity of neuronal loss in patient tissues. ND classifications were based on the following standard deviation limits: increased density *z* > 2SD, normal density *z* < 2SD, mild loss *z* < –2SD, moderate loss *z* < –3SD and severe loss z < –4SD. *Z*‐scores could not be calculated for patient tissues with complete loss of neuronal subtypes.

### Quadruple immunofluorescence to visualise OXPHOS complexes within interneurons

Quadruple immunofluorescence was performed to identify the nuclear DNA‐encoded complex I subunit (NADH:ubiquinone oxidoreductase subunit B8; NDUFB8) and mtDNA‐encoded complex IV subunit I (cytochrome *c* oxidase I; COXI) within mitochondria, using porin (mitochondrial outer membrane protein, VDAC1) as a mitochondrial mass marker, in parvalbumin+ and calretinin+ interneurons. Loss of NDUFB8 and COXI protein levels has previously been reported in Alpers' syndrome GABAergic interneurons, indicative of decreased enzymatic activities of complexes I and IV [14].

Prolonged fixation of tissues in formalin is known to alter the antigenicity of target epitopes; therefore, only tissues which were fixed in formalin for less than 1 year were used for immunofluorescence experiments (unpublished observation; supporting information Table S1). The immunofluorescence protocol has previously been described [14] but briefly involved heat‐mediated antigen retrieval in EDTA and blocking sections in 10% normal goat serum for 1 h at room temperature and blocking in Avidin and Biotin for 15 min each. Primary antibodies were diluted optimally in Tris‐buffered saline, 0.1% Tween 20® (TBST, pH 7.4), and were applied to the sections overnight at 4°C (supporting information Table S3). The following day, a biotinylated mouse IgG1 antibody was applied for 30 min at room temperature to amplify the signal of the NDUFB8 antibody. Appropriate Alexa Fluor‐conjugated secondary antibodies specific to the isotypes of the primary antibodies were applied to the sections for 2 h at 4°C (supporting information Table S3). Sections were finally incubated in 3.0% Sudan Black B for 10 min to minimise autofluorescence and were mounted in ProLong™ Gold antifade reagent to preserve fluorescent signals.

### Confocal microscopy

Immunofluorescent sections were imaged using an inverted ZEISS LSM800 confocal microscope and ZEISS ZEN (blue edition) software. A brightfield preview scan at x5 magnification was used to identify the same cortical region in which neuronal densities were quantified. A second preview scan was captured at x20 magnification (405 nm laser) to identify interneurons for image capture. The tiled image was then used as a map to select individual interneurons to be imaged at x63 magnification (oil immersion lens) using a x2.5 electronic zoom and two times line averaging. Image capture settings were optimised per experiment and were maintained for all cases. Single stained primary antibody control sections were used to check for channel bleed‐through. Approximately 50 parvalbumin+ and 50 calretinin+ interneurons were imaged on separate sections per case. For patient tissues with severe neuronal loss, all interneurons were captured within the region of interest.

### Image analysis

Immunofluorescent images were analysed using ZEISS ZEN Blue Desk software. An intensity threshold for the 405 nm channel was set to automatically outline the soma of individual interneurons. Within each interneuron, the mean optical intensities of the 405 nm (Parvalbumin/Calretinin), 488 nm (COXI), 546 nm (NDUFB8) and 647 nm (Porin) channels were measured. The ratio of log‐transformed NDUFB8 and COXI intensity values relative to log‐transformed Porin intensity values was calculated per interneuron, to normalise the abundance of NDUFB8 and COXI subunits relative to mitochondrial mass, thus ensuring any observed differences reflected changes in OXPHOS subunits rather than altered abundance of mitochondria. Using control OXPHOS ratios (NDUFB8/Porin, COXI/Porin), *z*‐scores were calculated to determine the severity of NDUFB8 and COXI deficiencies in patient interneurons. The following standard deviation limits were used as follows: increased expression z > 2SD, normal expression *z* < 2SD, low expression *z* < –2SD, deficiency z < –3SD and severe deficiency z < –4SD.

### Statistical analysis

All statistical analysis was performed using GraphPad Prism 9.0 (GraphPad Software, Inc., La Jolla, California) and R (R Core Team, 2020). The assumption of normality was assessed by visual inspection of Q‐Q plots and the Shapiro–Wilk test. Linear regression models were used to analyse ND data, total brain weights and porin data, with comparisons across groups by least square means, adjusted for multiple comparisons using the Tukey method. Immunofluorescence OXPHOS data were analysed using the Kruskal–Wallis test followed by Dunn's method for multiple comparisons or the Mann–Whitney *U* test. The level of significance (alpha value) was set at 0.05.

## RESULTS

### Clinical presentation

We investigated a postmortem cohort of eight patients with clinically and neuropathologically defined Alpers' syndrome and six patients with genetically confirmed Alpers' syndrome and POLG‐related encephalopathy (Table 1). This included 11 patients who had onset of symptoms in early infancy or childhood and three patients with an initial presentation in adolescence or early adult life, reflecting the bimodal onset of POLG‐related disease. Patient 1 through to Patient 8 had particularly rapid disease progressions with fatal consequences in infancy or childhood.

**TABLE 1:** Clinical and genetic details for the patients with Alpers' syndrome included in the current study

Case	Sex	Age: Onset	Age: Death	Seizures/status epilepticus	Developmental delay/regression	Cortical visual impairment	Liver dysfunction/failure/pathology	Cerebellar ataxia / hypotonia/hemiplegia	Electroencephalogram (EEG) findings	Bi‐allelic pathogenic POLG variants		Family history	Previously published
										cDNA	Protein		
P01^a^	M	2 m	5.5 m	+	+	+		+	Slow base rhythm, frontal delta waves, generalised spikes.	Unknown	Unknown	Affected sister	14
P02^a^	M	4 m	13 m	+	+	+	+	+	Hypsarrhythmia.	Unknown	Unknown		14
P03	F	11 m	14 m	+	+		+	+	Widespread irregular alpha and theta, moderate delta activity.	c.1399G > A/ c.2542G > A	p.[Ala467Thr]/ p.[Gly848Ser]		14
P04^a^	M	6 m	17 m	+	+		+		Unknown	Unknown	Unknown		
P05^a^	F	12 m	18 m	+	+	+	+	+	Unknown	Unknown	Unknown		
P06^a^	M	18 m	2.8y	+	+	+			Unknown	Unknown	Unknown	Affected sister	
P07^a^	F	2 m	4.0y	+	+	+			‘EEG confirmed epilepsy’.	Unknown	Unknown		14
P08^a^	F	6 m	7.0y	+	+	+	+	+	Occipital slow spike wave variants; dominant delta activity in fronto‐parietal regions; asymmetric dysrhythmia.	c.2243G > C/ c.2243G > C/	p.[Trp748Ser]/ p.[Trp748Ser]		14,30
P09	M	2y	11.9y	+	+	+	+	+	Posterior dominant rhythm of 5 Hz over both occipital lobes. Frequent posterior intermittent delta polymorphic activity of 2.5–3 Hz.	c.1399G > A/ c.2542G > A	p.[Ala467Thr]/ p.[Gly848Ser]	Affected brother	
P10^a^	M	6y	12.5y	+		+		+	Widespread irregular slow wave activity.	Unknown	Unknown		14
P11^a^	F	6 m	14.0y	+	+	+		+	Abnormal, isoelectric activity.	Unknown	Unknown	Affected brother	14
P12	F	18.0y	23.0y	+		+	+	+	Continuous right posterior spike‐and‐wave activity.	c.1399G > A/ c.1399G > A	p.[Ala467Thr]/ p.[Ala467Thr]		31
P13	F	20.0y	24.0y	+		+		+	Encephalopathic, right posterior quadrant spike waves.	c.1399G > A/ c.2243G > C	p.[Ala467Thr]/ p.[Trp748Ser]		15,24,32, 33, 34, 35
P14	F	16.0y	28.0y	+		+		+	Unknown	c.1399G > A/ c.2243G > C	p.[Ala467Thr]/ p.[Trp748Ser]	Affected brother	

*Note*: Clinical reports for patients with Alpers' syndrome have been summarised where available. Suitable tissues were unavailable for sequencing the common pathogenic *POLG* variants, and extraction of sufficient DNA from formalin‐fixed, paraffin‐embedded tissues was unsuccessful*. POLG* RefSeq: NM_002693.2.

Abbreviations: m, months; y, years.

^a^ Historical patients who died prior to the identification of pathogenic variants in *POLG* known to cause Alpers' syndrome.

Intractable epilepsy was a salient feature in all 14 patients and included focal myoclonic and tonic–clonic seizures, generalised seizures and status epilepticus. Seizures were often preceded by visual auras, headaches, vomiting and fever. All patients with Alpers' syndrome displayed signs of developmental delay and/or developmental regression. Visual impairments, including a lack of visual attentiveness, were the next most commonly reported symptom in 12 patients. Seven patients showed signs of hepatic dysfunction and/or hepatic pathology, including hepatic failure in four patients. However, permission was not provided to assess the liver at postmortem for all patients. Seven patients had an ataxic phenotype suggestive of cerebellar dysfunction.

### Neuropathological features

The neuropathological features observed within the occipital, frontal and temporal cortices of the patients with Alpers' syndrome are summarised by Table 2. The total weight of patient brains was significantly lower than control (*p* = 0.002) and SUDEP patient (*P* < 0.001) brain weights, indicating severe, generalised cerebral atrophy in Alpers' syndrome (supporting information Figure S1). Focal necrotic lesions, characterised by extensive neuronal loss, reactive astrogliosis, spongiosis and thinning of the cortical ribbon, show predilection for the occipital cortex [12]. Neuropathological changes including extensive neuronal loss were also prominent within the frontal cortex, while the temporal cortex was the least severely affected cortical region analysed.

**TABLE 2:** Neuropathological and neuroimaging summary

Patient	Brain weight (g)	Macroscopic external neuropathology	Microscopic neuropathology			Neuroimaging findings
			Occipital cortex	Frontal cortex	Temporal cortex	
P01^a^	Unknown	Unknown	Atrophic gyri and severe thinning of white matter	Unknown	Unknown	CT: Symmetrical hydrocephalus
P02^a^	Unknown	Unknown	Atrophic and gliotic gyri	Areas of focal neuronal loss and ischaemia; thinned cortical ribbon.	Unknown	Unknown
P03	860	Brain appeared oedematous.	Mild spongiform changes	Preserved	Mild spongiform changes	CT: Mild generalised cerebral atrophy.
P04^a^	380	Microcephaly, severe generalised cerebral atrophy and gliosis	Severe necrosis with few surviving neurons; extensive astrogliosis.	Severe necrosis with few surviving neurons; extensive astrogliosis.	Less severe necrosis of the medial temporal lobe, with more surviving neurons; extensive astrogliosis	MRI: Gross cerebral atrophy; high signal in the pons (but normal at postmortem)
P05^a^	580	Cerebral atrophy predominantly involving the occipital and parietal lobes; thin cerebellar folia; ventricular enlargement	Severe necrosis with few surviving neurons; loss of the Line of Gennari; extensive astrogliosis	Severe necrosis with few surviving neurons; extensive astrogliosis	Severe necrosis with few surviving neurons; extensive astrogliosis	CT: Severe atrophy with development of prominence of the ventricular system and extensive periventricular lucencies
P06^a^	490	Severe generalised cortical atrophy; severe cortical thinning (<1 mm in depth) affecting the entire frontal and parietal lobes, and most of the occipital and temporal lobes	Severe necrosis, most severely affected layer III; extensive astrogliosis	Severe necrosis and devastated layer III; extensive astrogliosis	Severe necrosis predominantly affecting layer III; extensive astrogliosis	Air encephalogram showed cerebral atrophy
P07^a^	563.1	Microcephaly, cerebral atrophy and thinned cortical ribbon, most severe in the occipital poles. Marked ventricular dilatation of the occipital lobe	Severe necrosis with few surviving neurons, most severely affecting layers II–III; extensive astrogliosis; secondary white matter changes	Marked neuronal loss, thinned cortical ribbon	Marked neuronal loss; preserved cortical ribbon	Unknown
P08^a^	Unknown	Severe diffuse atrophy; marked internal and external hydrocephalus; thinned cortical ribbon and white matter; preserved cerebellum	Widespread spongy degeneration; profound neuronal loss most severely affecting layers III and V; extensive astrogliosis; secondary white matter changes	Widespread spongy degeneration; profound neuronal loss and astrogliosis; white matter changes	Unknown	Unknown
P09	Unknown	Unknown	Moderate neuronal loss; moderate astrogliosis	Relatively preserved neuronal density	Relatively preserved neuronal density	Unknown
P10^a^	1057	Atrophied occipital lobes; atrophied cerebellar hemispheres	Severe widespread cortical necrosis typically affecting gyral crests; extensive white matter damage	Neuronal loss and astrogliosis	Unknown	CT: Progressive cerebral atrophy; ventricular enlargement
P11^a^	264	Severe atrophy; 3–4 mm thinned gyri	Most extensive area of atrophy; near‐total loss of neurons	Marked atrophy of the cortex with near‐total loss of neurons; white matter changes	Unknown	CT: Marked symmetrical atrophy
P12	1263	Oedematous brain and thinned cortical ribbon, most severely affected primary visual cortex; atrophied cerebellar grey matter	Severe focal necrotic lesion affecting all layers; extensive astrogliosis and vacuolation; loss of myelin and gliosis of the white matter	Mild to moderate neuronal loss	Mild to moderate neuronal loss	MRI: Bilateral occipital and right parietal SLE. Right occipital, right temporal and right parietal atrophy
P13	Unknown	Atrophied frontal lobe and mild cerebral atrophy	Severe focal necrotic lesion; extensive neuronal loss most severely affecting layers III, V and VI; extensive astrogliosis	Thinned layers III, V and VI; vascular pathology; focal ischaemic‐like changes	Thinned layers III, V and VI; white matter shows mildly thickened vessels	MRI: Left occipital, left parietal and thalamic SLE. Left thalamus and medulla seizure‐related changes
P14	1352	Mild occipital demyelination around the ventricle	Severe focal necrotic lesion; widespread severe neuronal loss and extensive astrogliosis	Marked neuronal loss; cytoplasmic swelling	Marked neuronal loss; cytoplasmic swelling	MRI: Bilateral occipital, bilateral parietal, right frontal, left frontal SLE. Left occipital atrophy

*Note*: Neuropathological findings have previously been described for Patient 1 to Patient 3, Patient 7 to Patient 8, Patient 10 to Patient 12 and Patient 13. [14, 15, 30]

Abbreviations: CT, computed tomography; MRI, magnetic resonance imaging; SLE, stroke‐like episode (focal cortical lesion).

^a^ Historical patients with limited neuropathological data.

### Loss of cortical inhibitory interneurons and pyramidal neurons

Quantification of interneuron densities revealed topographical differences in the relative abundance of particular interneuron subtypes. The occipital cortex had the highest density of parvalbumin+ interneurons relative to the frontal (*P* < 0.001) and temporal (*P* < 0.001) cortices (supporting information Figure S2). Within the occipital cortex, the mean density of parvalbumin+ interneurons was also higher than all other interneuron subtypes quantified.

**FIGURE 1:**
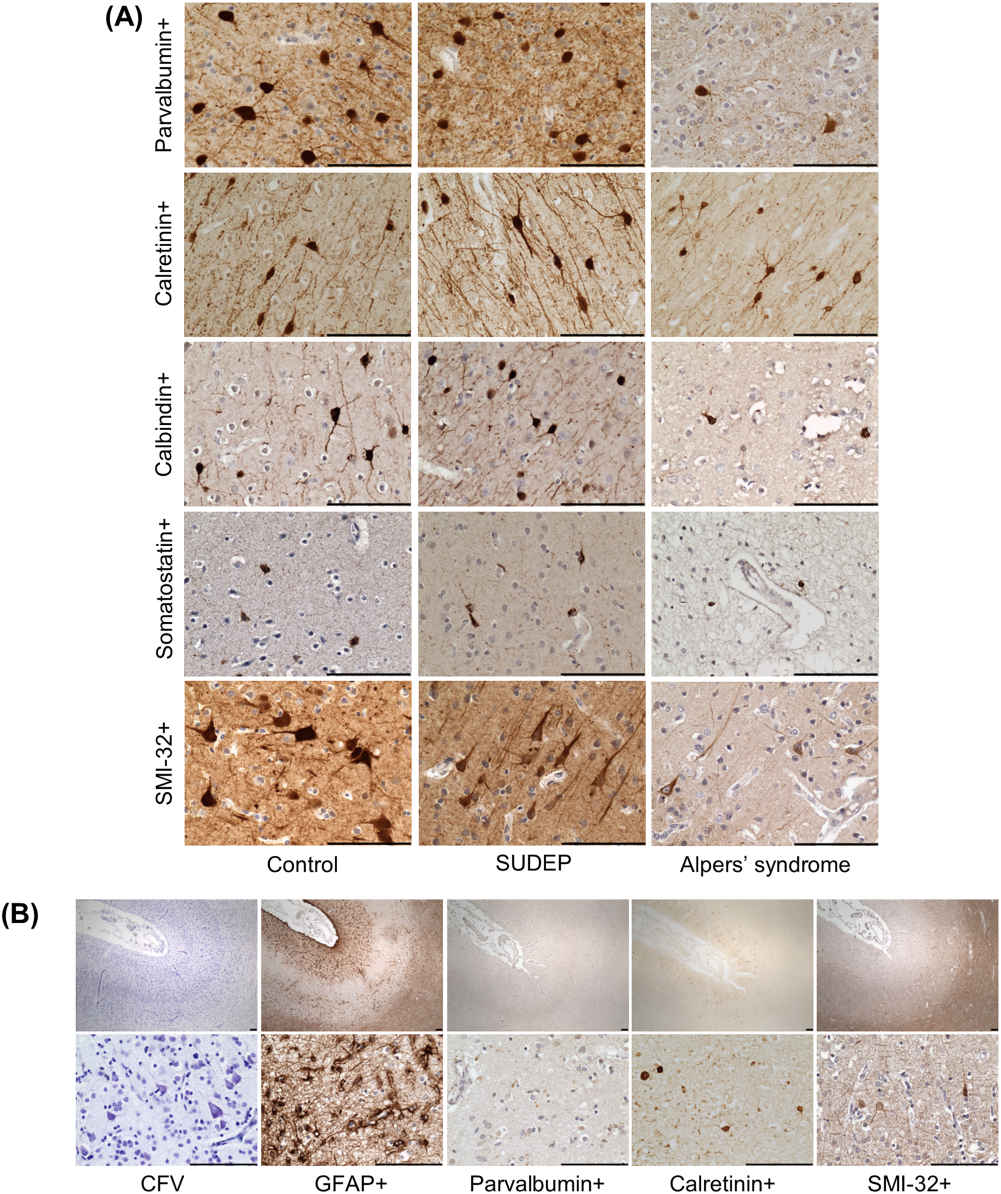
Interneuron and pyramidal neuron loss in the primary visual cortex in Alpers' syndrome. (A) Representative images showing a marked loss of parvalbumin+, calbindin+, somatostatin+ interneurons and SMI‐32+ pyramidal neurons and a preservation of calretinin+ interneurons, within the occipital cortex of patients with Alpers' syndrome, relative to controls and sudden unexpected death in epilepsy (SUDEP) patients. (B) A focal stroke‐like lesion within the primary visual cortex of P13 shows severe neuronal drop out (Cresyl fast violet; CFV), extensive reactive astrogliosis (glial fibrillary acidic protein; GFAP+), a complete loss of parvalbumin+ interneurons and a severe loss of SMI‐32+ pyramidal neurons. Calretinin+ interneurons are preserved within the superficial cortical layers of the necrotic lesion. Scale bars = 100 μm.

**FIGURE 2:**
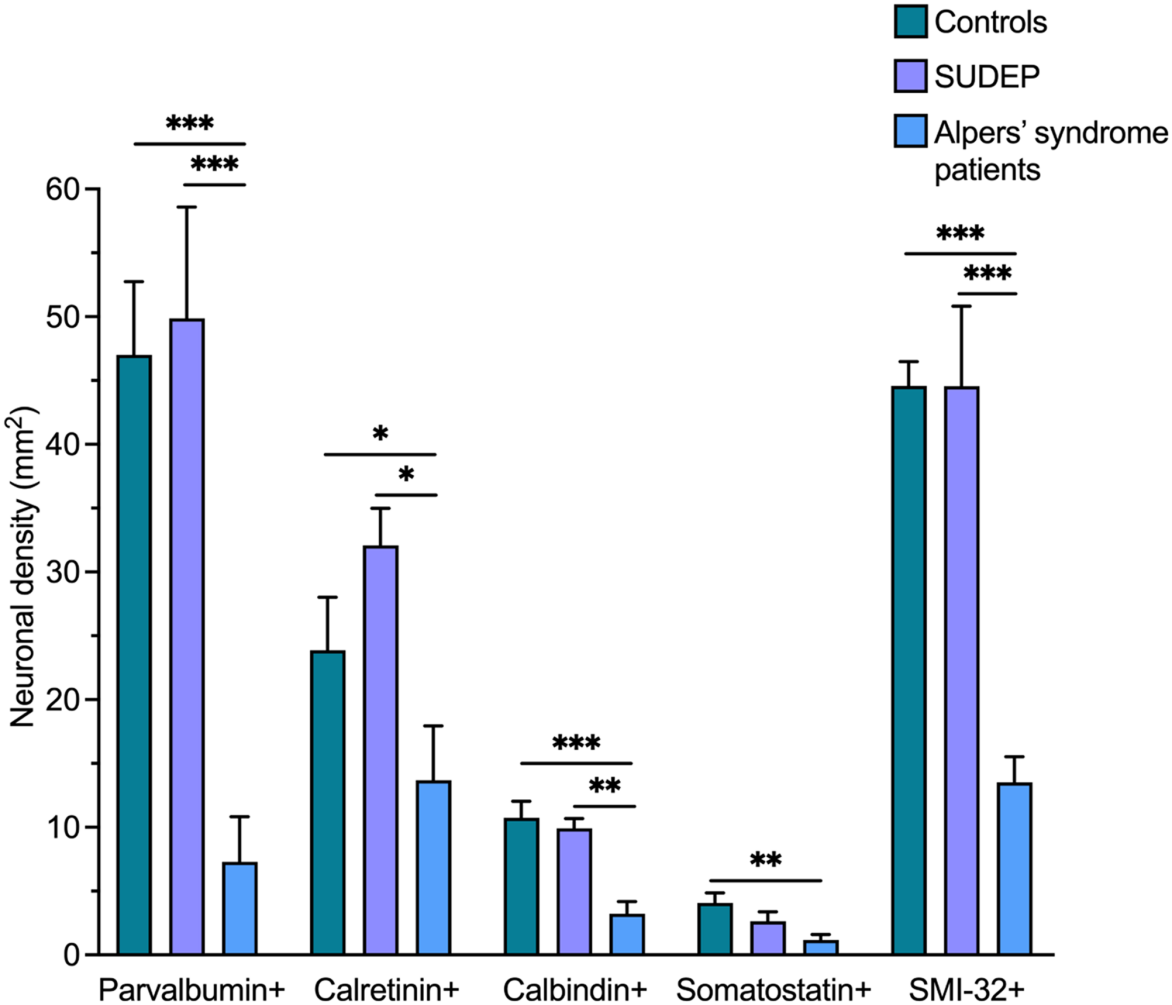
Decreased neuronal densities in the primary visual cortex in Alpers' syndrome. Densities of parvalbumin+, calretinin+, calbindin+, somatostatin+ interneurons and SMI‐32+ pyramidal neurons within the primary visual cortex of patients with Alpers' syndrome (*N =* 10), compared with neuronal densities in control (*N =* 8) and sudden unexpected death in epilepsy (SUDEP) (*N =* 5) tissues, are presented (mean ± standard error). Compared with control densities, 15% of parvalbumin+ interneurons remain in Alpers' syndrome tissues (group level analysis), vs ~30% of calbindin+ interneurons, somatostatin+ interneurons and SMI‐32+ pyramidal neurons, and 57% of calretinin+ interneurons. Data analysed using a linear regression model: *** *P* < 0.001, ** *P* < 0.01, * *P* < 0.05

Representative images of the morphology and density of neuronal subtypes quantified within the occipital cortex are presented in Figure 1. All patients demonstrated variably reduced interneuron densities in all cortical regions analysed. Interneuron loss was most pronounced within the occipital cortex, irrespective of focal necrotic lesions. Quantification revealed a significant loss of parvalbumin+ interneurons within the occipital cortex of patients with Alpers' syndrome relative to controls and SUDEP patients (*P* < 0.001, Figure 2) including a severe loss in 8 of 10 patients (*z* < −4). At the group level, parvalbumin+ interneuron densities were also significantly reduced in the frontal cortex (*P* < 0.001) and temporal cortex (*P* < 0.05) of patients with Alpers' syndrome relative to controls (supporting information Figure S3). The density of calbindin+ and somatostatin+ interneurons was also variably decreased, particularly in the occipital and frontal cortices (Figure 2 and supporting information Figure S3). However, the percentage of parvalbumin+ interneurons remaining in Alpers' syndrome patient tissues compared with control tissues (15%) was consistently lower than the proportion of remaining calbindin+ and somatostatin+ interneurons (30%), indicating an increased degeneration of parvalbumin+ interneurons in Alpers' syndrome (Figure 2).

Interestingly, calretinin+ interneurons were the least affected interneuron subtype, and were only significantly reduced in the occipital cortex of patients with Alpers' syndrome relative to controls at the group level (*P* < 0.05, Figure 2) but were preserved in many patient tissues (*z* > −2), even within cortex affected by focal lesions with a total loss of parvalbumin+ interneurons (Figure 1B). Patient tissues which were affected by widespread necrosis also frequently showed normal, or mildly decreased, calretinin+ interneuron density despite a total loss of parvalbumin+ and calbindin+ interneurons.

As comparison, pyramidal neuron densities were also quantified and demonstrated lower densities within the occipital cortex of patients with Alpers' syndrome relative to controls and SUDEP patients (*P* < 0.001, Figure 2), whereas pyramidal neuron densities were only severely reduced in the frontal (*P* < 0.01) and temporal (*P* > 0.05) cortices of patients who had an early‐onset, rapidly progressive phenotype of Alpers' syndrome (supporting information Figure S3). Albeit the overall loss of pyramidal neurons as a percentage was consistently lower than the loss of parvalbumin+ interneurons.

### Severe OXPHOS deficiencies are observed in parvalbumin+ interneurons

Since there was a consistent, severe loss of parvalbumin+ interneurons in Alpers' syndrome, immunofluorescence was used to interrogate OXPHOS protein expression in these cells to provide further insight into the aetiology of this interneuron vulnerability. The mean optical intensity of complex I and complex IV subunits, NDUFB8 and COXI, respectively, in conjunction with mitochondrial mass marker porin, was quantified within parvalbumin+ interneurons within each cortical region.

**FIGURE 3:**
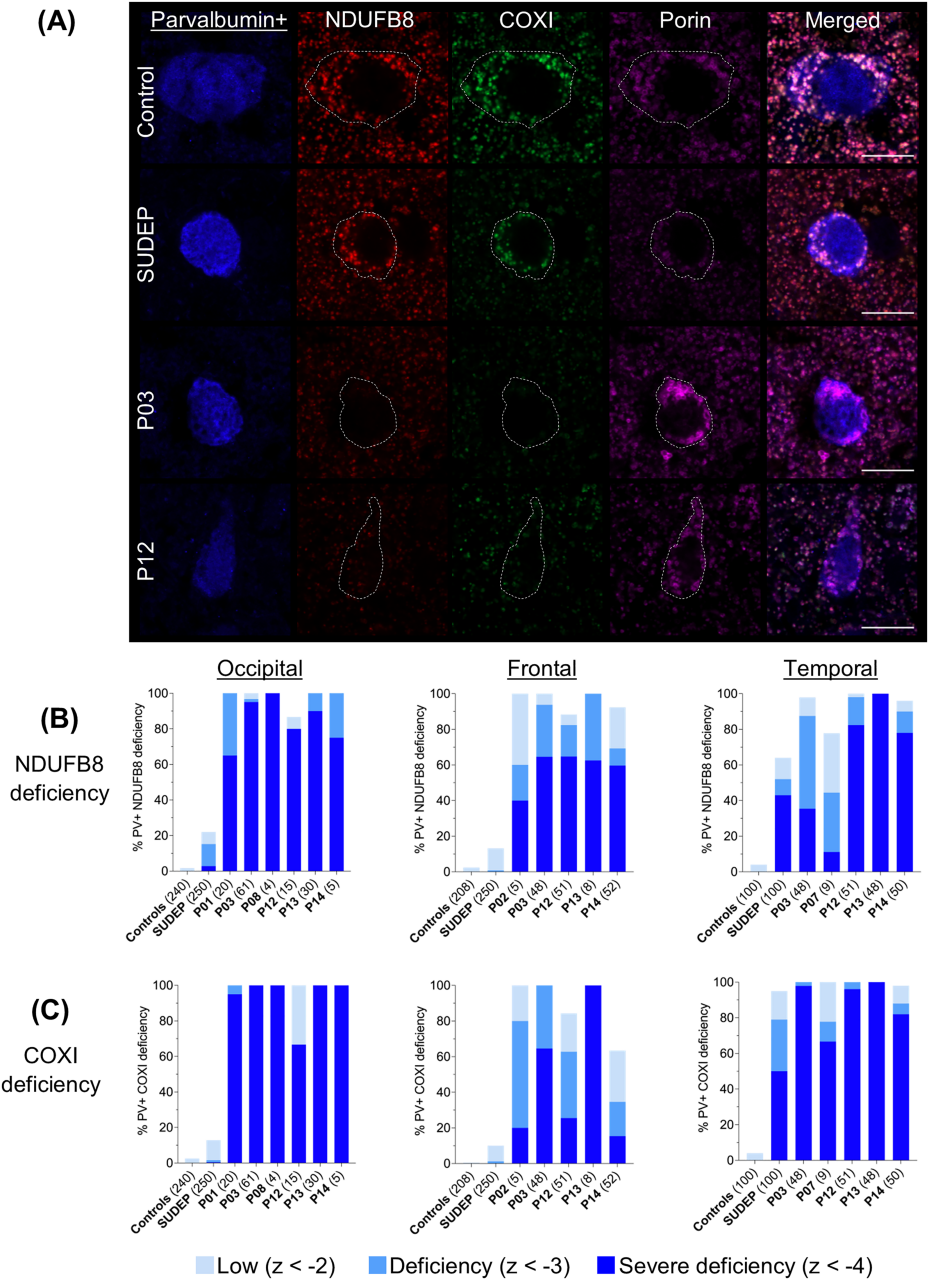
Severe oxidative phosphorylation (OXPHOS) deficiencies within parvalbumin+ interneurons in Alpers' syndrome. (A) Severe loss of OXPHOS complex I (NDUBF8) and complex IV (COXI) subunits within mitochondria (porin) within parvalbumin+ interneurons of the primary visual cortex in patients with Alpers' syndrome. Patient parvalbumin+ interneurons also show increased intensities of porin. Scale bars = 10 μm. (B,C) Parvalbumin+ somas were automatically detected based on 405 nm+ signal. Quantification of mean optical intensities of (B) NDUFB8 and (C) COXI relative to porin was quantified within parvalbumin+ interneurons of the occipital, frontal and temporal cortex that revealed a high percentage of severe OXPHOS deficiencies in patients with Alpers' syndrome. Total number of parvalbumin+ interneurons analysed per group and per patient are presented in brackets. Number of control cases (occipital: *N =* 5, frontal: *N =* 4, temporal, *N =* 2) and sudden unexpected death in epilepsy (SUDEP) cases (occipital: *N =* 5, frontal: *N =* 5, temporal, *N =* 2)

Patient tissues showed markedly reduced immunoreactivity of both NDUFB8 and COXI within parvalbumin+ interneurons, despite preserved or increased porin. Quantification of mean optical intensities revealed a high proportion of parvalbumin+ interneurons from all patients with Alpers' syndrome had combined severe NDUFB8 and COXI deficiencies (Figure 3A–C). In the occipital cortex, there was a significant main effect across groups (Kruskal–Wallis, *P* < 0.0001), with post hoc analyses revealing significantly reduced levels of NDUFB8 and COXI in all patients with Alpers' syndrome and the SUDEP patient group relative to controls (*P* < 0.0001), albeit most SUDEP patient *z*‐scores were within the normal range (Figure 3B,C) indicating intact NDUFB8 and COXI protein levels. Severe deficiencies were also observed in the temporal cortex and to a lesser extent, within the frontal cortex in Alpers' syndrome (Figure 3B,C). Interestingly, parvalbumin+ interneurons from patients with Alpers' syndrome showed a trend towards greater loss of COXI protein expression relative to NDUFB8. This may suggest that there is a preferential impairment of complex IV within parvalbumin+ interneurons in Alpers' syndrome.

The mean optical intensity of porin was significantly increased in Alpers' syndrome parvalbumin+ interneurons (*n =* 135) compared with control interneurons (*n =* 240) (*P* < 0.01) (supporting information Figure S4), with most patient parvalbumin+ interneurons of the occipital cortex harbouring a porin *z*‐score > 2 indicating increased mitochondrial mass (supporting information Figure S5), whereas the majority of parvalbumin+ interneurons within the frontal and temporal cortices in Alpers' syndrome showed normal or reduced levels of porin relative to controls (z < 2) (supporting information Figure S5).

In all cortical regions, there was a significant main effect of the mean optical intensity of parvalbumin+ calcium‐binding protein (CBP) across all groups (Kruskal–Wallis, *P* < 0.05) with post hoc analyses revealing a significant loss of parvalbumin+ intensity within the majority of Alpers' syndrome patient parvalbumin+ interneurons relative to controls (*P* < 0.05) (supporting information Figure S6). Since parvalbumin expression is activity‐regulated [36, 37], this may suggest reduced activity of parvalbumin+ interneurons in Alpers' syndrome.

### Milder OXPHOS deficiencies are observed in calretinin+ interneurons

Since calretinin+ interneurons demonstrated more intact neuronal densities in Alpers' syndrome, the immunofluorescence assay was adapted to directly compare OXPHOS protein expression in these cells.

**FIGURE 4:**
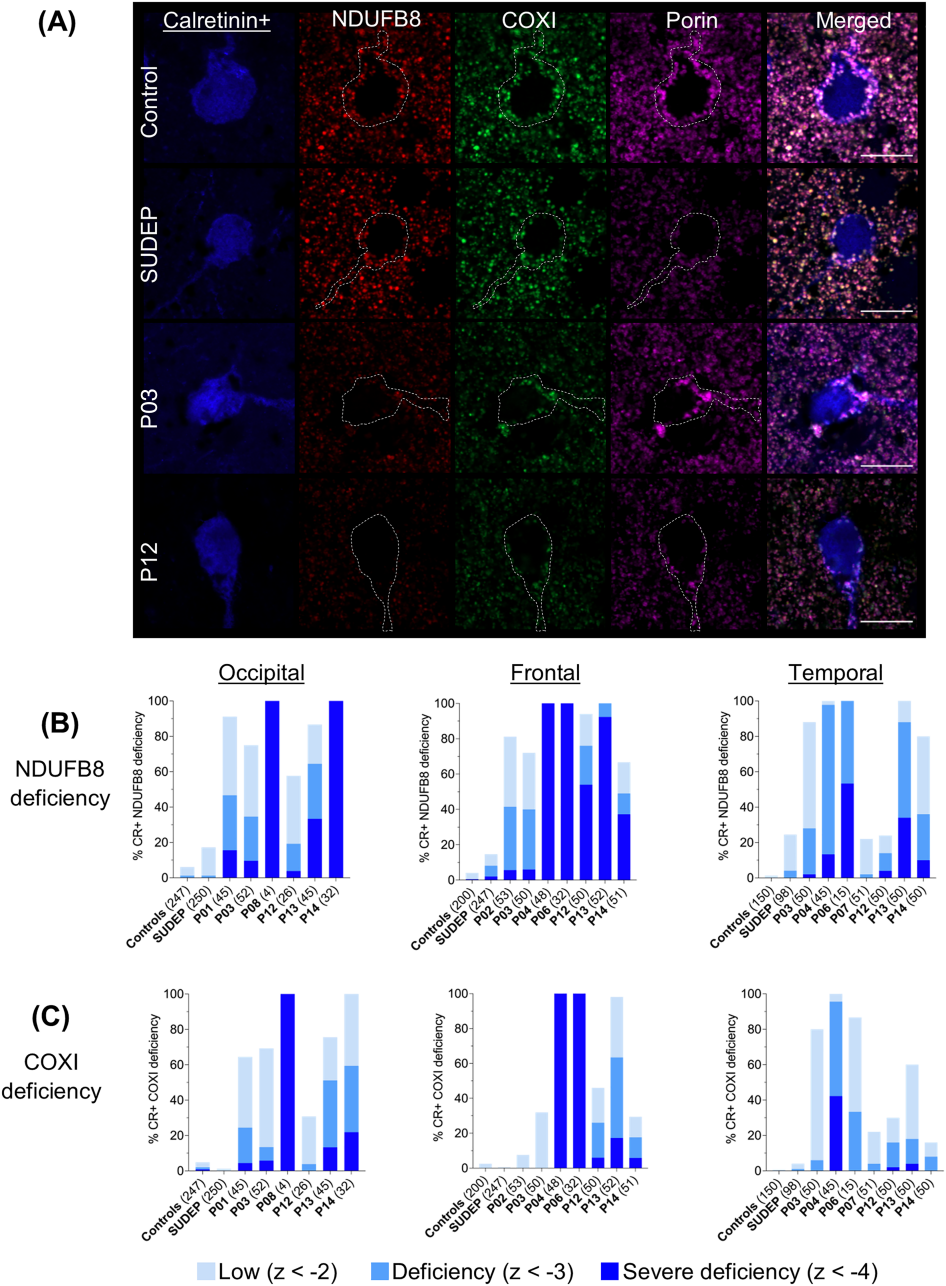
Variable levels of oxidative phosphorylation (OXPHOS) deficiencies within calretinin+ interneurons in Alpers' syndrome. (A) Variable loss of OXPHOS complex I (NDUBF8) and complex IV (COXI) subunits within mitochondria (porin) within calretinin+ interneurons of the primary visual cortex in patients with Alpers' syndrome. Scale bars = 10 μm. (B) Calretinin+ somas were automatically detected based on 405 nm+ signal. Quantification of the mean optical intensities of (B) NDUFB8 and (C) COXI relative to porin, within calretinin+ interneurons of the occipital, frontal and temporal cortex revealed low‐to‐severe OXPHOS deficiencies in patients with Alpers' syndrome. Total number of calretinin+ interneurons analysed per group and per patient are presented in brackets. Number of control cases (occipital: *N =* 5, frontal: *N =* 4, temporal, *N =* 3) and sudden unexpected death in epilepsy (SUDEP) cases (occipital: *N =* 5, frontal: *N =* 5, temporal, *N =* 2)

Calretinin+ interneurons from Alpers' syndrome patient tissues displayed surprisingly low levels of NDUFB8 immunoreactivity and to a lesser extent decreased immunoreactivity of COXI within mitochondria compared with control interneurons (Figure 4A). Analysis of calretinin+ interneurons revealed a significant main effect across all groups in the occipital, frontal and temporal cortices (Kruskal–Wallis, *P* < 0.0001), with post hoc analyses demonstrating significantly decreased levels of NDUFB8 and COXI within calretinin+ interneurons from patients with Alpers' syndrome relative to controls (*P* < 0.0001) (Figure 4B,C). A higher proportion of Alpers' syndrome patient calretinin+ interneurons showed more severe NDUFB8 deficiencies relative to COXI deficiencies, suggesting a more severe dysfunction of complex I. There was also a trend towards more severe NDUFB8 and COXI deficiencies in calretinin+ interneurons from necrotic tissues vs preserved tissues.

The levels of NDUFB8 and COXI deficiencies within calretinin+ interneurons were significantly less severe when compared with parvalbumin+ interneurons in the occipital and temporal cortices of almost all patients with Alpers' syndrome (Mann–Whitney, *P* < 0.05). Furthermore, all patients with Alpers' syndrome showed more severe COXI deficiencies within parvalbumin+ interneurons relative to calretinin+ interneurons in the frontal cortex (Mann–Whitney, *P* < 0.0001). This suggests a consistent severe dysfunction of parvalbumin+ interneurons relative to calretinin+ interneurons in Alpers' syndrome, even in tissues affected by severe neuronal necrosis.

At the group level, calretinin+ interneurons from patients with Alpers' syndrome harboured a significantly higher mean optical intensity of porin relative to control interneurons (*P* < 0.001) (supporting information Figure S4); however, mean patient porin *z*‐scores were mostly within the normal range (*z* < 2) indicating normal mitochondrial mass (supporting information Figure S5). Moreover, calretinin+ interneurons from patients with Alpers' syndrome did not show a consistent loss of calretinin+ CBP, with most patients showing either normal levels or increased levels of calretinin+ intensity relative to controls (supporting information Figure S6). Collectively, these findings indicate less severe affectation of calretinin+ interneurons in Alpers' syndrome.

### Increased c‐fos immunoreactivity indicates cortical hyperactivity

To investigate cortical hyperactivity within the primary visual cortex in Alpers' syndrome, immunohistochemistry to identify c‐fos+ cells was performed. *FOS* is an immediate early gene which encodes the transcription factor c‐fos and is transiently upregulated in response to neuronal activity [38]. Therefore, elevated c‐fos abundance could be considered an indirect marker of hyperactive neurons prior to death.

**FIGURE 5:**
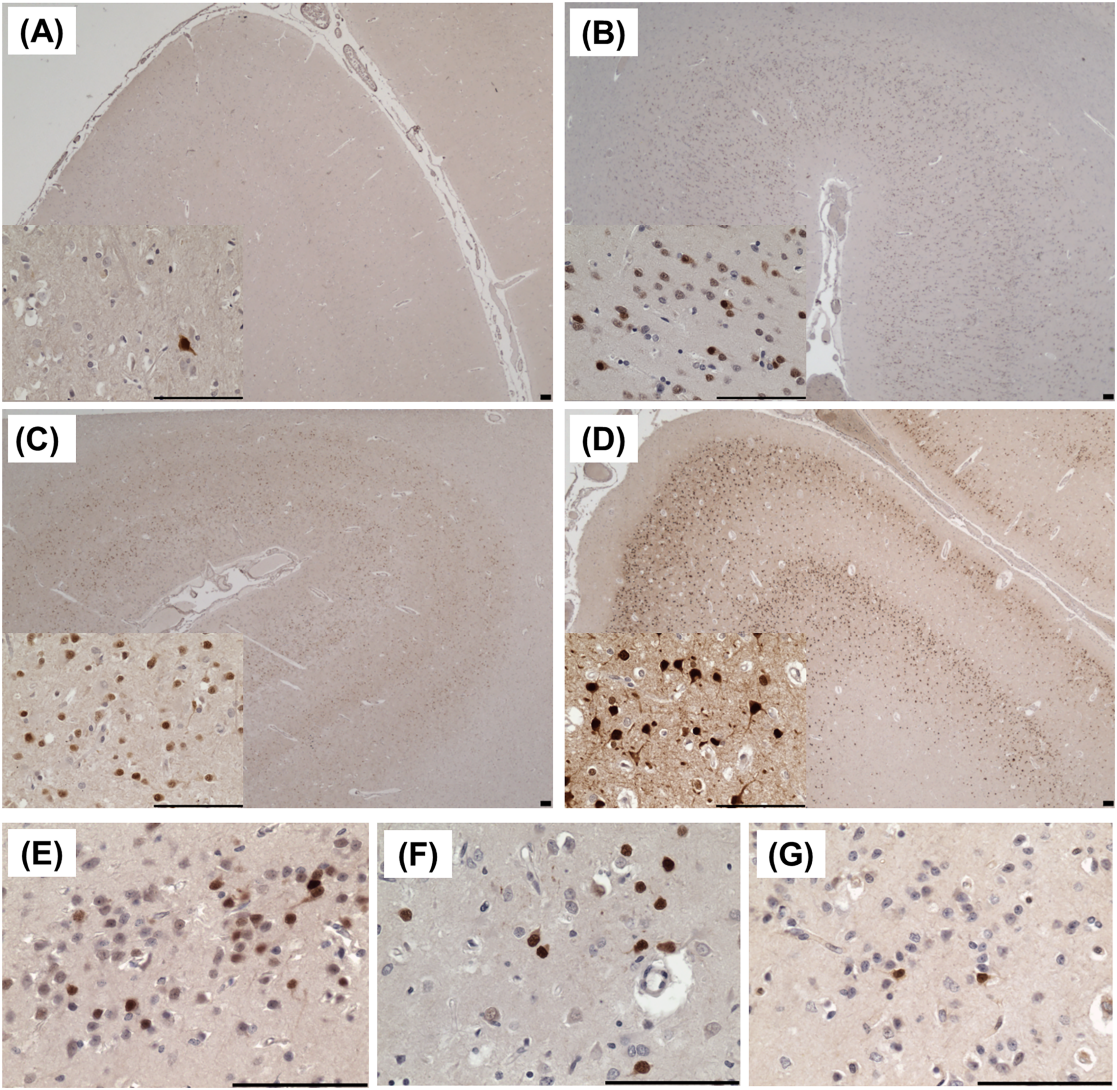
Neuronal hyperactivity in the primary visual cortex in Alpers' syndrome. C‐fos immunoreactivity within the primary visual cortex of (A) controls, (B) sudden unexpected death in epilepsy (SUDEP) patients and (C–G) patients with Alpers' syndrome. (C) A focal necrotic cortical lesion in P14 shows detectable levels of c‐fos in all cells which morphologically appear to be glial cells. (D) Preserved adjacent occipital cortex tissues in P14 show intense c‐fos staining in all neuronal and glial cells. (E–G) other patient tissues show small clusters of c‐fos+ cells throughout the occipital cortex. Scale bars = 100 μm.

Control tissues showed low levels of c‐fos+ immunoreactivity as expected (Figure 5A), whereas a higher density of c‐fos+ cells was observed in tissues from patients with SUDEP (Figure 5B) and Alpers' syndrome (Figure 5C–G). Within a focal cortical stroke‐like lesion of Patient 14, most cells expressed detectable levels of c‐fos (Figure 5C). Based on morphological features of these cells, and considering the near complete neuronal loss within the lesion, the c‐fos+ immunoreactivity appears to localise to glial cells. In adjacent occipital cortex tissue where neuronal densities are intact, all neuronal cells express intense levels of c‐fos (Figure 5D), indicating extensive neuronal hyperactivity; this may be associated with recent seizure events and death in status epilepticus. Occipital cortex tissues from other patients with Alpers' syndrome displayed small clusters of c‐fos+ cells which may represent the few remaining neurons which were metabolically active (Figure 5E–G).

## DISCUSSION

Intractable epilepsy is a defining feature of patients with Alpers' syndrome, and previous studies have demonstrated an extensive involvement of the inhibitory interneuron population which might be important for the generation of seizures in these patients [14, 15]. We performed a detailed neuropathological investigation to explore the susceptibility of specific subtypes of cortical interneurons in 14 patients with Alpers' syndrome and demonstrated a consistent, severe loss of parvalbumin+ interneurons and severe OXPHOS deficiencies within remaining parvalbumin+ interneurons. This was in contrast to calretinin+ interneurons, which harboured milder OXPHOS deficits and greater resilience to neurodegeneration. Furthermore, we have demonstrated enrichment of parvalbumin+ interneurons in the occipital cortex which, combined with their apparent increased vulnerability, suggests that this underlies the occipital focus of seizures in Alpers' syndrome.

### Parvalbumin+ interneuron loss shows a predilection for the occipital cortex

The primary visual cortex is the predominant site of epileptogenesis in Alpers' syndrome; therefore, it is not surprising that neurodegenerative changes are most profound within the occipital lobes [6]. We quantified the densities of specific cortical neuronal subtypes to better understand how altered neuronal densities may contribute to epileptogenesis in Alpers' syndrome. Cortical neuronal loss within occipital cortex tissues from patients with Alpers' syndrome involved a marked loss of a number of cell types, of which parvalbumin+ interneurons were the most severely affected. Within control occipital cortex tissues, parvalbumin+ interneurons were present at a much higher density than in frontal and temporal cortices, suggesting that parvalbumin+ interneurons fulfil an important regulatory role, limiting excitatory activity and regulating physiological cortical rhythms, within the occipital lobe. Consequently, this may predispose the primary visual cortex to seizure activity in patients with Alpers' syndrome due to the metabolic vulnerability and subsequent loss of these network‐regulating cells. This may help explain the predominantly occipital focus of seizures in Alpers' syndrome and POLG‐related encephalopathy. Interestingly, calretinin+ interneurons were consistently the least affected interneuron subtype, even in regions of severe neuronal necrosis. There were no clear patterns to the loss of calbindin+ and somatostatin+ interneurons; however, the densities were low in control tissues suggesting that a loss of these interneuron subtypes may be less detrimental to neuronal networks. Alternatively, the low densities may reflect a lack of neural redundancy suggesting that the loss of these interneuron subtypes may have a more severe impact.

Neuronal loss was not confined to the occipital cortices in this Alpers' syndrome patient cohort. Profound interneuron and pyramidal neuron loss occurred in the frontal cortex of almost all patients and within the temporal cortex of patients who had an early‐onset of symptoms and died in childhood. The widespread loss of pyramidal neurons in these patients may reflect more severe seizure activity and rapidly progressive neurodegeneration. Although perhaps infrequently reported, frontal lobe involvement is common in patients with Alpers' syndrome, and neuronal loss within the dorsolateral prefrontal cortex may be associated with cognitive decline, cognitive disinhibition and anxiety in patients with Alpers' syndrome [39, 40]. The overall preservation of neuronal densities in the SUDEP group suggests that seizure‐associated neurodegeneration is not common within these cortical regions in SUDEP, albeit the cohort was small.

### Parvalbumin+ interneurons harbour severe OXPHOS deficiencies

In addition to the consistent loss of parvalbumin+ interneurons and overall preservation of calretinin+ interneurons in Alpers' syndrome, we demonstrated differential OXPHOS deficiency profiles within these two interneuron subtypes. This involved a variably decreased expression of complex I and complex IV subunits, likely to impair not only ATP generation within these cells but also inhibitory neurotransmission, leading to a reduction in the threshold for seizures. Increased oxidative stress and disturbed calcium handling, associated with mitochondrial dysfunction, may also contribute to seizure susceptibility in Alpers' syndrome.

Interestingly, parvalbumin+ interneurons frequently showed a greater loss of complex IV subunits relative to complex I subunits, particularly within the younger Alpers' syndrome patients. This novel finding suggests a more severe involvement of complex IV in parvalbumin+ interneuron dysfunction in Alpers' syndrome. However, complex I deficiency exceeded complex IV deficiency within calretinin+ interneurons. A disproportionate loss of complex I subunits is a frequent histopathological finding in other neuronal subtypes in mitochondrial diseases [12, 14, 15, 41], Parkinson's disease [42, 43], Lewy body dementia [44] and TLE [45]. Since parvalbumin+ interneuron loss was extensive in Alpers' syndrome patient tissues, the severe loss of complex IV subunits may be more detrimental to the functioning and survival of parvalbumin+ interneurons. Complex IV‐mediated dysfunction of parvalbumin+ interneurons may also underlie the vulnerability and impairment of these interneurons in other neurodevelopmental disorders associated with mitochondrial dysfunction, including autism spectrum disorder (ASD) and schizophrenia [46, 47, 48] and may also explain why epilepsy is a common comorbidity of these disorders [49, 50].

A proportion of interneurons from SUDEP patients also harboured variably reduced levels of NDUFB8 and COXI protein levels, albeit less severe than the patients with Alpers' syndrome. Mitochondrial dysfunction has previously been reported in patients with TLE [45, 51]. While mitochondrial dysfunction is the primary pathology underlying interneuron vulnerability and driving seizure activity in Alpers' syndrome, glutamatergic‐induced neurotoxicity may underlie the secondary dysfunction of mitochondria in TLE and SUDEP [52, 53].

### Increased resilience of calretinin+ interneurons

An apparent vulnerability of parvalbumin+ interneurons in Alpers' syndrome is perhaps an unsurprising finding due to the well‐recognised dependence of parvalbumin+ interneurons on functioning mitochondria [21, 22, 54]. However, the relative preservation of calretinin+ interneurons in patient tissues affected by severe neuronal necrosis was remarkable. Multiple studies have demonstrated a reduced vulnerability of calretinin+ interneurons to glutamatergic‐mediated excitotoxicity, including preservation of calretinin+ interneurons in the sclerotic hippocampi of TLE patients, increased resilience of calretinin+ interneurons exposed to excessive calcium and glutamate levels in vitro and a preservation of calretinin+ interneurons in the knock‐in amyloid precursor protein mouse model associated with neuronal hyperexcitability [55, 56, 57]. This suggests a decreased neuropathological involvement of calretinin+ interneurons in neurodegenerative diseases and common epilepsies characterised by excitotoxicity. The nonfast spiking activity of calretinin+ interneurons is likely to be associated with reduced metabolic requirements compared with parvalbumin+ interneurons, which may underlie the increased resilience of calretinin+ interneurons to mitochondrial dysfunction in Alpers' syndrome. However, some calretinin+ interneurons preferentially target calretinin‐negative GABAergic interneurons in human cortical tissues, albeit a lower proportion compared with mouse cortex [58, 59]. Therefore, it is tempting to speculate that the preservation of superficial calretinin+ interneurons within Alpers' syndrome patient tissues may further impair the inhibitory response to neuronal hyperexcitability through inhibition of remaining interneurons.

### Altered calcium buffering capacity and activity of parvalbumin+ interneurons

CBP have a critical role in buffering intracellular calcium ions at presynaptic and postsynaptic sites [60, 61]. Alpers' syndrome patient parvalbumin+ interneurons show reduced levels of parvalbumin+ protein expression relative to control interneurons which may be indicative of reduced calcium‐binding capacity. During seizure activity, this may exacerbate the calcium overload resulting from neuronal hyperexcitability and extensive mitochondrial dysfunction, contributing to the increased vulnerability of parvalbumin+ interneurons. Alpers' syndrome patients also showed a consistent increase in mitochondrial mass within parvalbumin+ interneurons of the occipital cortex which may represent a compensatory response to severe OXPHOS deficiencies. A loss of parvalbumin+ protein expression and altered mitochondrial mass has been reported in ASD, suggesting that overlapping mechanisms may mediate parvalbumin+ interneuron dysfunction in ASD and Alpers' syndrome [46, 47].

It is tempting to speculate that parvalbumin+ protein expression may be reduced in an activity‐dependent manner due to metabolic failure arising from extensive OXPHOS deficiencies and reduced excitatory input from pyramidal neurons, due to severe pyramidal neuron loss in Alpers' syndrome [36, 37], whereas the majority of patient calretinin+ interneurons showed normal or increased levels of calretinin+ protein suggesting preserved activity of calretinin+ interneurons and intact calcium buffering capacity, which may partly underlie the increased resilience of these interneurons.

### SLE are seizure‐mediated events

We have provided further evidence of seizure‐associated activity mediating SLE in patients with Alpers' syndrome. Neuronal energy failure due to severe OXPHOS deficiency is hypothesised to underlie the almost complete neuronal loss associated with stroke‐like lesions [13, 15]. We have demonstrated a near‐total loss of inhibitory interneurons and an upregulation of c‐fos within almost all neurons and glial cells within cortex affected by a recent SLE, suggesting hyperactivity of these cells. This supports the hypothesis of neuronal hyperexcitability and seizures precipitating SLE in patients with Alpers' syndrome and POLG‐related encephalopathy [13, 15].

### Future perspectives

Our neuropathological investigations have provided a detailed anatomical insight to the vulnerability of interneuron subtypes in end‐stage disease tissues affected by severe epilepsy and encephalopathy in Alpers' syndrome. Future studies involving a larger Alpers' syndrome patient cohort, an array of mitochondrial disease encephalopathies and nonprimary mitochondrial epilepsies should be performed to further explore the differential vulnerability of parvalbumin+ and calretinin+ interneurons by investigating changes in mtDNA integrity and quantity within these interneurons. Functional validation studies using appropriate in vitro disease models recapitulating the neuropathological features of mitochondrial epilepsy, including parvalbumin+ interneuron vulnerability, should also be performed to overcome the limitations of using postmortem tissues to determine the cause and effect of parvalbumin+ interneuron dysfunction and degeneration [32]. Suitable models could then be utilised to test novel therapies which improve the functioning of parvalbumin+ interneurons and consider the interactions with nonneuronal cells including astrocytes which potentially have a neuropathological role in POLG‐related disease [32, 62, 63]. It is likely that novel treatments targeting mitochondria to prevent the dysfunction of these highly important organelles and to preserve the functioning of parvalbumin+ interneurons may benefit an array of patients with common epilepsies and neurodevelopmental disorders.

## CONCLUSION

We have demonstrated a severe dysfunction and degeneration of inhibitory interneurons in cortical tissues from patients with Alpers' syndrome which likely underlies the loss of inhibitory neurotransmission mediating seizure activity in patients. Seizures will subsequently increase the energy demands of already metabolically compromised interneurons, further exacerbating the cycle of neuronal hyperexcitability and seizure‐associated neurodegeneration. Our novel findings of parvalbumin+ interneuron vulnerability vs calretinin+ interneuron resilience may occur in other types of mitochondrial encephalopathies, epilepsies and neurodevelopmental disorders associated with mitochondrial dysfunction. Modelling mitochondrial dysfunction within parvalbumin+ interneurons may provide a suitable system for testing novel therapeutics to ameliorate the seizure‐associated dysfunction and degeneration of parvalbumin+ interneurons.

## CONFLICT OF INTEREST

The authors declare no conflict of interest.

## ETHICS STATEMENT

The study was granted ethical approval for the use of postmortem tissues from BRAIN UK (19/SC/0217), NBTR (19/NE/0008), EBTB (East of Scotland Research Ethics Service REC1) and NBB. The Brain banks are human tissue authority‐approved and informed consent was obtained.

## AUTHOR CONTRIBUTIONS

LAS conducted experiments and acquired data for the study. LAS and AB performed statistical analyses. LAS, DE, NZL, RM and RWT contributed to the design of the study and interpretation of data. All authors have critically revised the manuscript and gave their final approval.

### PEER REVIEW

The peer review history for this article is available at https://publons.com/publon/10.1111/nan.12833.

## Supporting information


**Supplementary Figure S1** **Decreased brain weight in Alpers' syndrome.** Symbols indicate total brain weights of individuals in each group. The total weight of brains from patients with Alpers' syndrome is significantly lower compared to age‐matched controls (Linear regression model, *P* < 0.01) and SUDEP patients (*P* < 0.001). Linear regression line is plotted for each group.

**Supplementary Figure S2** **Topographic density of neuronal subtypes in control cortical tissue**. Densities of parvalbumin+, calretinin− + calbindin+, somatostatin+ interneurons and SMI‐32 + pyramidal neurons within the occipital, frontal and temporal cortices of control tissues. Mean neuronal densities (mm^2^) ± standard error of the mean (SEM) are presented. Number of controls per brain region: occipital N = 8; frontal N = 7; temporal N = 6. Neuronal density data was analysed using a linear regression model; *** *P* < 0.001, * *P* < 0.05.

**Supplementary Figure S3** **Neuronal loss in the frontal and temporal cortex in Alpers' syndrome**. Densities of parvalbumin+, calretinin+, calbindin+, somatostatin+ interneurons and SMI‐32 + pyramidal neurons in the **(A)** frontal cortex and **(B)** temporal cortex of patients with Alpers's syndrome, compared to densities in control and SUDEP patient tissues. Mean neuronal densities (mm^2^) ± standard error of the mean (SEM) are presented. Controls (frontal N = 7, temporal N = 6), SUDEP patients (frontal N = 5, temporal N = 2) and patients with Alpers' syndrome (frontal
N = 9, temporal N = 9). Neuronal density data was analysed using a linear regression model; *** *P* < 0.001, ** *P* < 0.01, * *P* < 0.05.

**Supplementary Figure S4** I**ncreased mitochondrial mass in Alpers' syndrome patient interneurons**. Porin z‐scores within parvalbumin+ (**PV**) and calretinin+ (**CR**) interneurons of the occipital, frontal and temporal cortices of control, SUDEP patient and Alpers' syndrome patient tissues are presented. Z‐score > 2 indicates an increased mean optical intensity of porin. Data analysed at the group level using a linear regression model: *** *P* < 0.001, ** *P* < 0.01.

**Supplementary Figure S5** **Porin protein levels in parvalbumin+ interneurons and calretinin+ interneurons in Alpers' syndrome.** Porin z‐scores within **(A)** patient parvalbumin+ and **(B)** calretinin+ interneurons of the **(i)** occipital, **(ii)** frontal and **(iii)** temporal cortices are presented as box and whisker plots; **+** indicates mean value. Data analysed using Kruskal‐Wallis followed by Dunn's method for multiple comparisons: *** *P* < 0.0001, * *P* < 0.05.

**Supplementary Figure S6** **Calcium‐binding proteins in Alpers' syndrome**. Mean optical intensity of **a** parvalbumin+ and **b** calretinin+ calcium‐binding proteins (CBP) within the **(i)** occipital, **(ii)** frontal and **(iii)** temporal cortices are presented as box and whisker plots; **+** indicates mean value. Data analysed using Kruskal‐Wallis followed by Dunn's method for multiple comparisons: *** *P* < 0.0001, ** *P* < 0.01, * *P* < 0.05.

**Supplementary Table S1.** Demographic details for patient and control tissues

**Supplementary Table S2.** Primary antibodies for chromogen immunohistochemistry experiments

**Supplementary Table S3.** Antibodies for quadruple immunofluorescence assay

## Data Availability

The data that support the findings of this study are available from the corresponding author upon reasonable request.
