# Supplementary figures and images for: Delineating selective vulnerability of inhibitory interneurons in Alpers' syndrome
Source: Neuropathol Appl Neurobiol. 2022 Jul 19;48(6):e12833. doi: 10.1111/nan.12833 (PMC9546160; doi:10.1111/nan.12833)

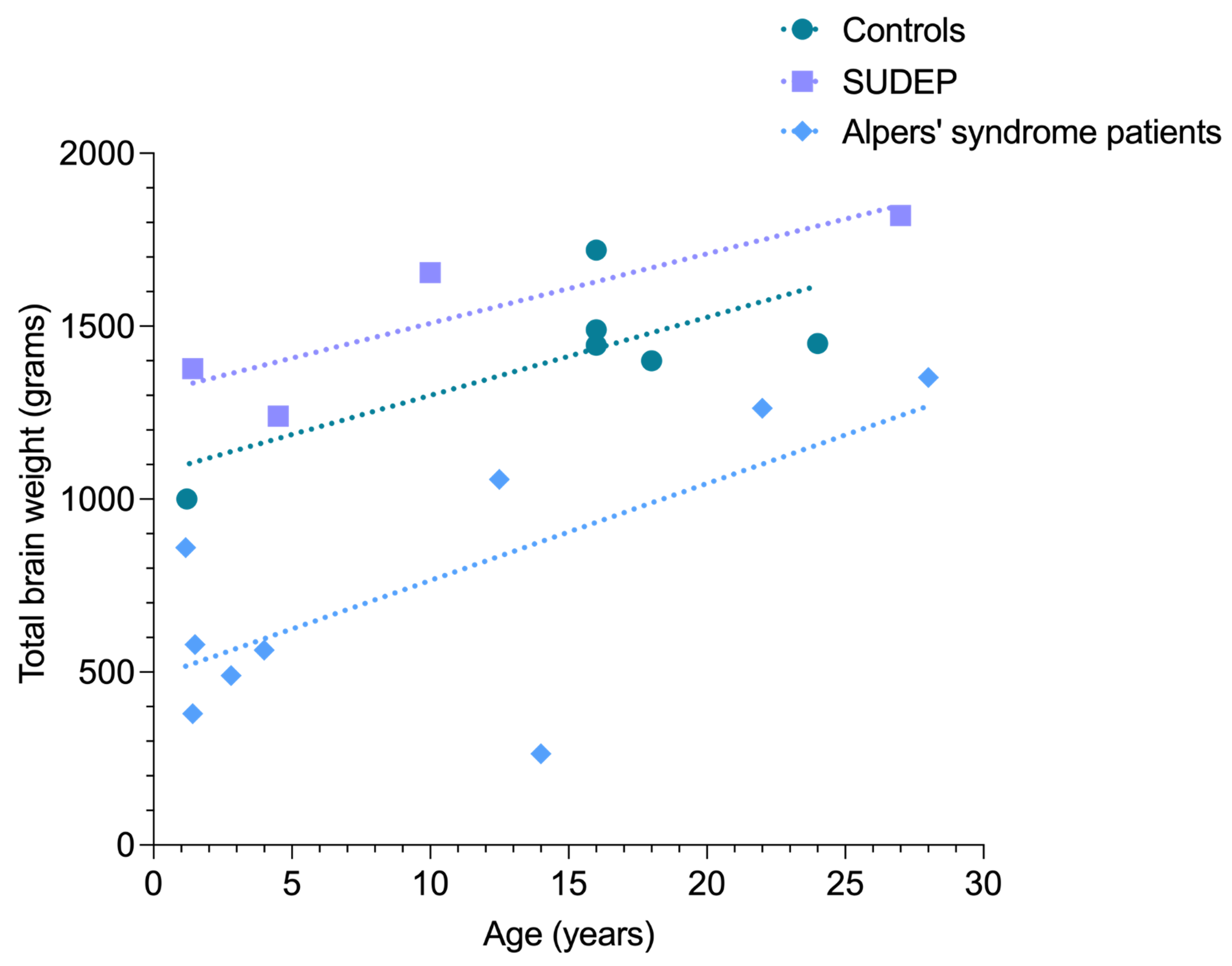

Supplement: Supplementary file 1 — Supplementary Figure S1 Decreased brain weight in Alpers' syndrome. Symbols indicate total brain weights of individuals in each group. The total weight of brains from patients with Alpers' syndrome is significantly lower compared to age‐matched controls (Linear regression model, P < 0.01) and SUDEP patients (P < 0.001). Linear regression line is plotted for each group. [file NAN-48-0-s004.tif]

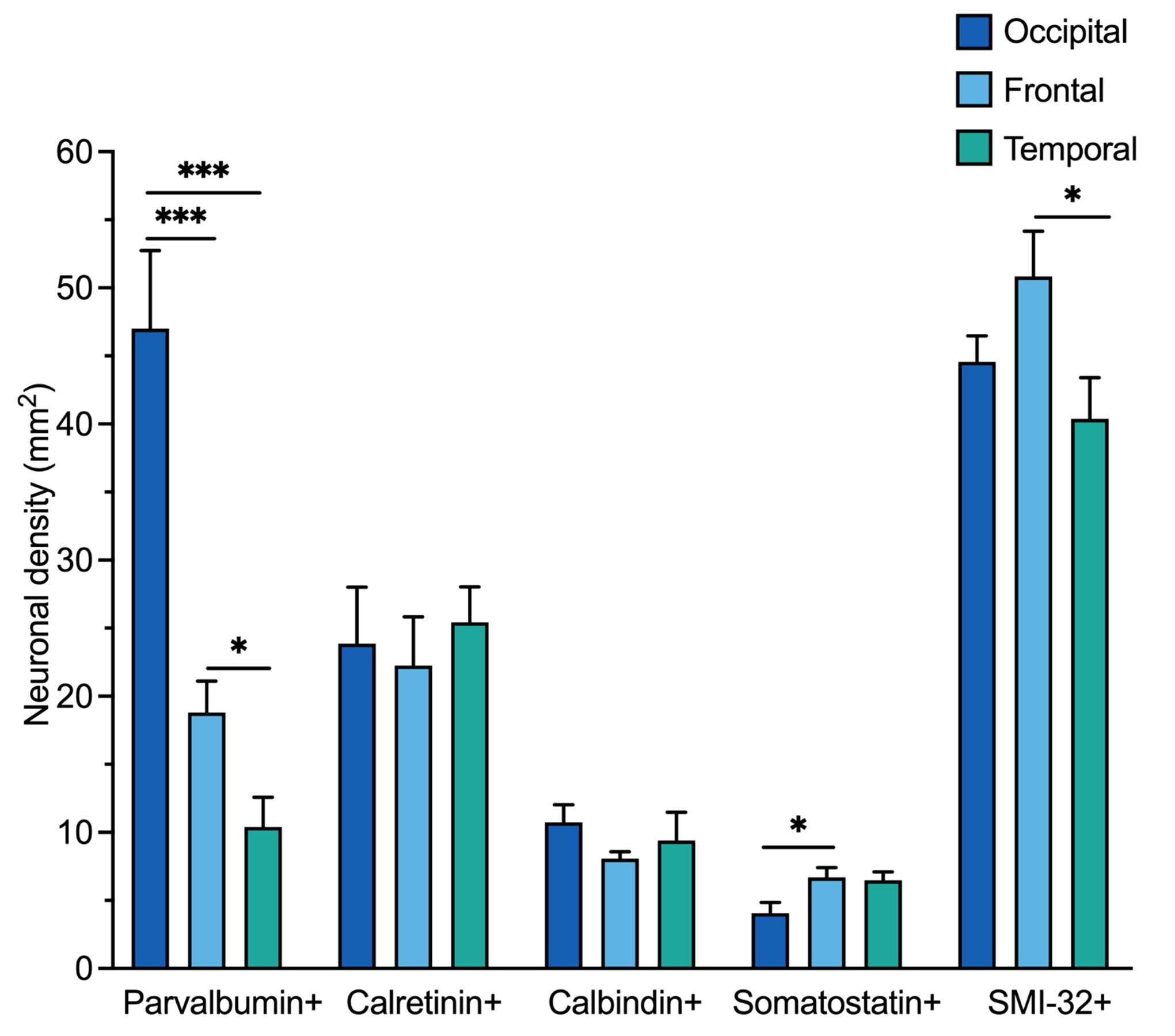

Supplement: Supplementary file 2 — Supplementary Figure S2 Topographic density of neuronal subtypes in control cortical tissue. Densities of parvalbumin+, calretinin− + calbindin+, somatostatin+ interneurons and SMI‐32 + pyramidal neurons within the occipital, frontal and temporal cortices of control tissues. Mean neuronal densities (mm2) ± standard error of the mean (SEM) are presented. Number of controls per brain region: occipital N = 8; frontal N = 7; temporal N = 6. Neuronal density data was analysed using a linear regression model; *** P < 0.001, * P < 0.05. [file NAN-48-0-s007.tif]

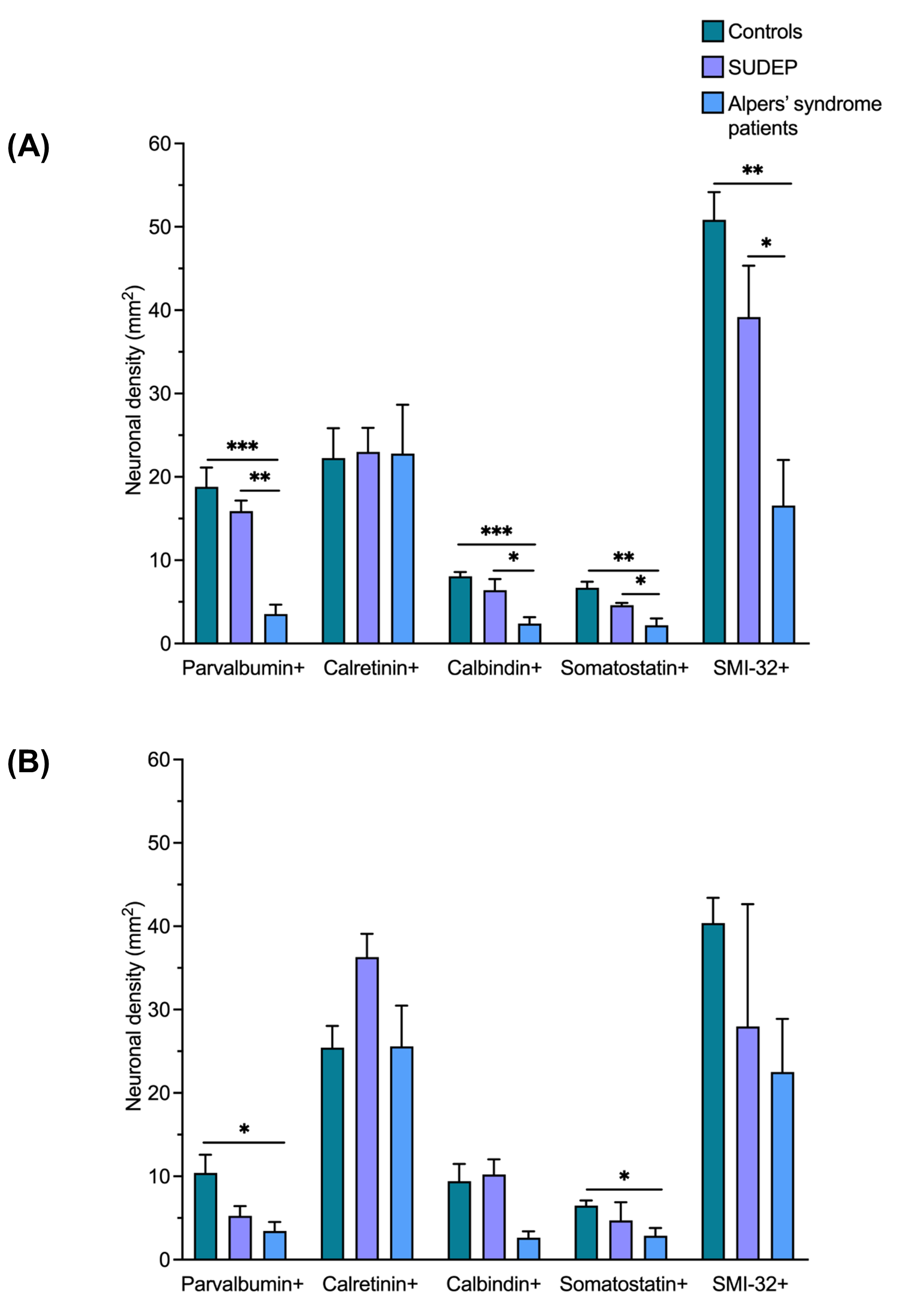

Supplement: Supplementary file 3 — Supplementary Figure S3 Neuronal loss in the frontal and temporal cortex in Alpers' syndrome. Densities of parvalbumin+, calretinin+, calbindin+, somatostatin+ interneurons and SMI‐32 + pyramidal neurons in the (A) frontal cortex and (B) temporal cortex of patients with Alpers's syndrome, compared to densities in control and SUDEP patient tissues. Mean neuronal densities (mm2) ± standard error of the mean (SEM) are presented. Controls (frontal N = 7, temporal N = 6), SUDEP patients (frontal N = 5, temporal N = 2) and patients with Alpers' syndrome (frontal N = 9, temporal N = 9). Neuronal density data was analysed using a linear regression model; *** P < 0.001, ** P < 0.01, * P < 0.05. [file NAN-48-0-s002.tif]

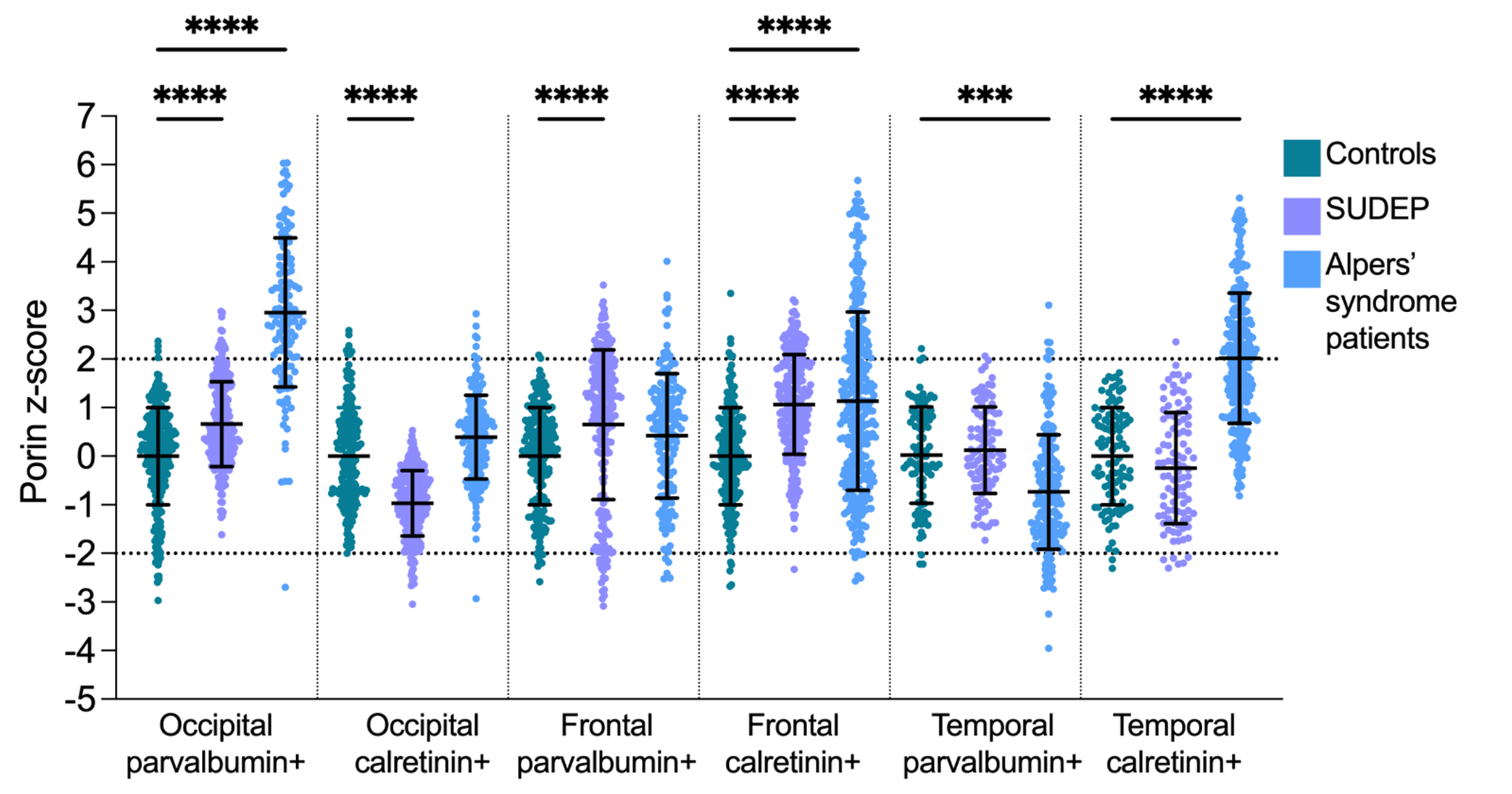

Supplement: Supplementary file 4 — Supplementary Figure S4 Increased mitochondrial mass in Alpers' syndrome patient interneurons. Porin z‐scores within parvalbumin+ (PV) and calretinin+ (CR) interneurons of the occipital, frontal and temporal cortices of control, SUDEP patient and Alpers' syndrome patient tissues are presented. Z‐score > 2 indicates an increased mean optical intensity of porin. Data analysed at the group level using a linear regression model: *** P < 0.001, ** P < 0.01. [file NAN-48-0-s009.tif]

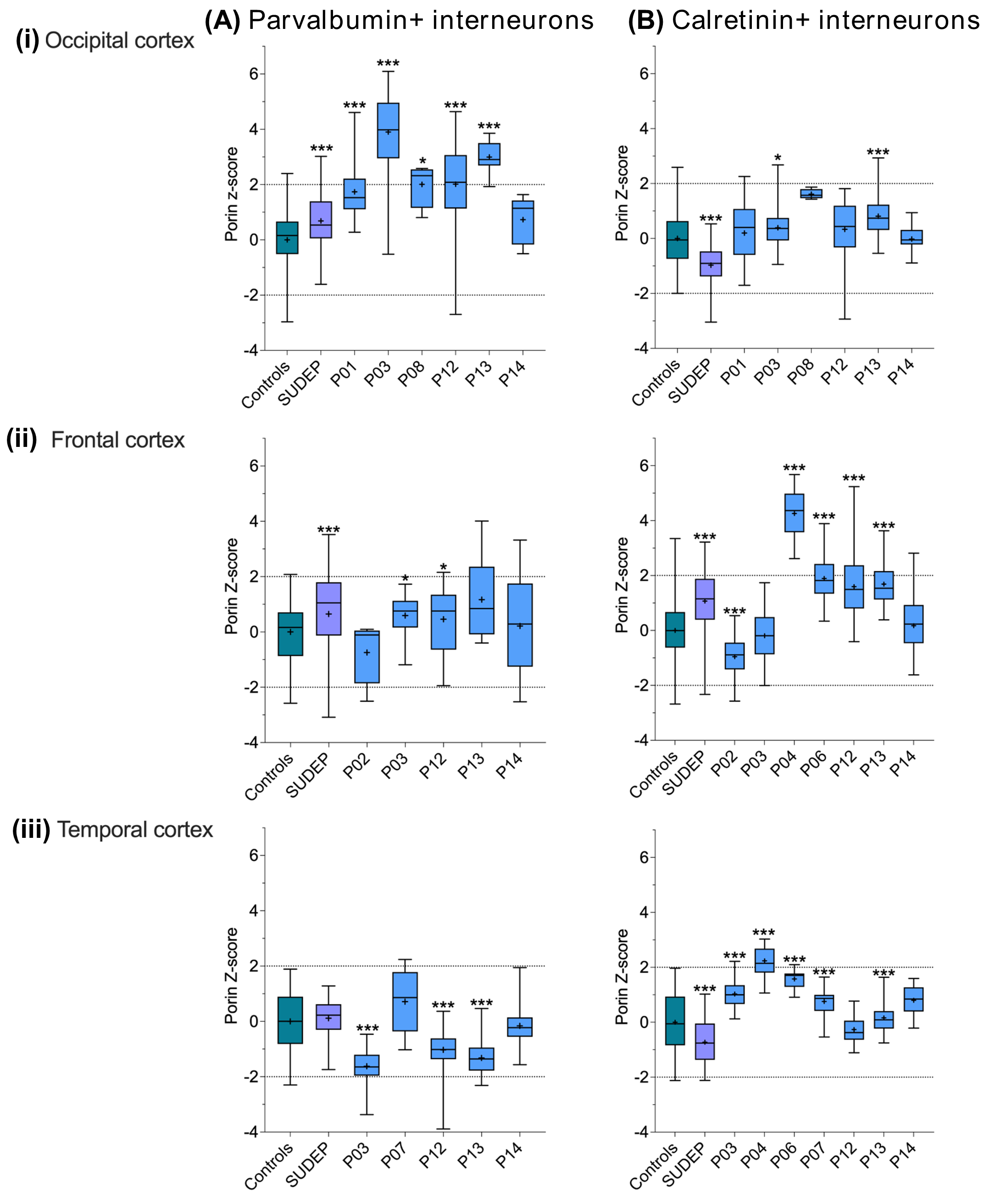

Supplement: Supplementary file 5 — Supplementary Figure S5 Porin protein levels in parvalbumin+ interneurons and calretinin+ interneurons in Alpers' syndrome. Porin z‐scores within (A) patient parvalbumin+ and (B) calretinin+ interneurons of the (i) occipital, (ii) frontal and (iii) temporal cortices are presented as box and whisker plots; + indicates mean value. Data analysed using Kruskal‐Wallis followed by Dunn's method for multiple comparisons: *** P < 0.0001, * P < 0.05. [file NAN-48-0-s006.tif]

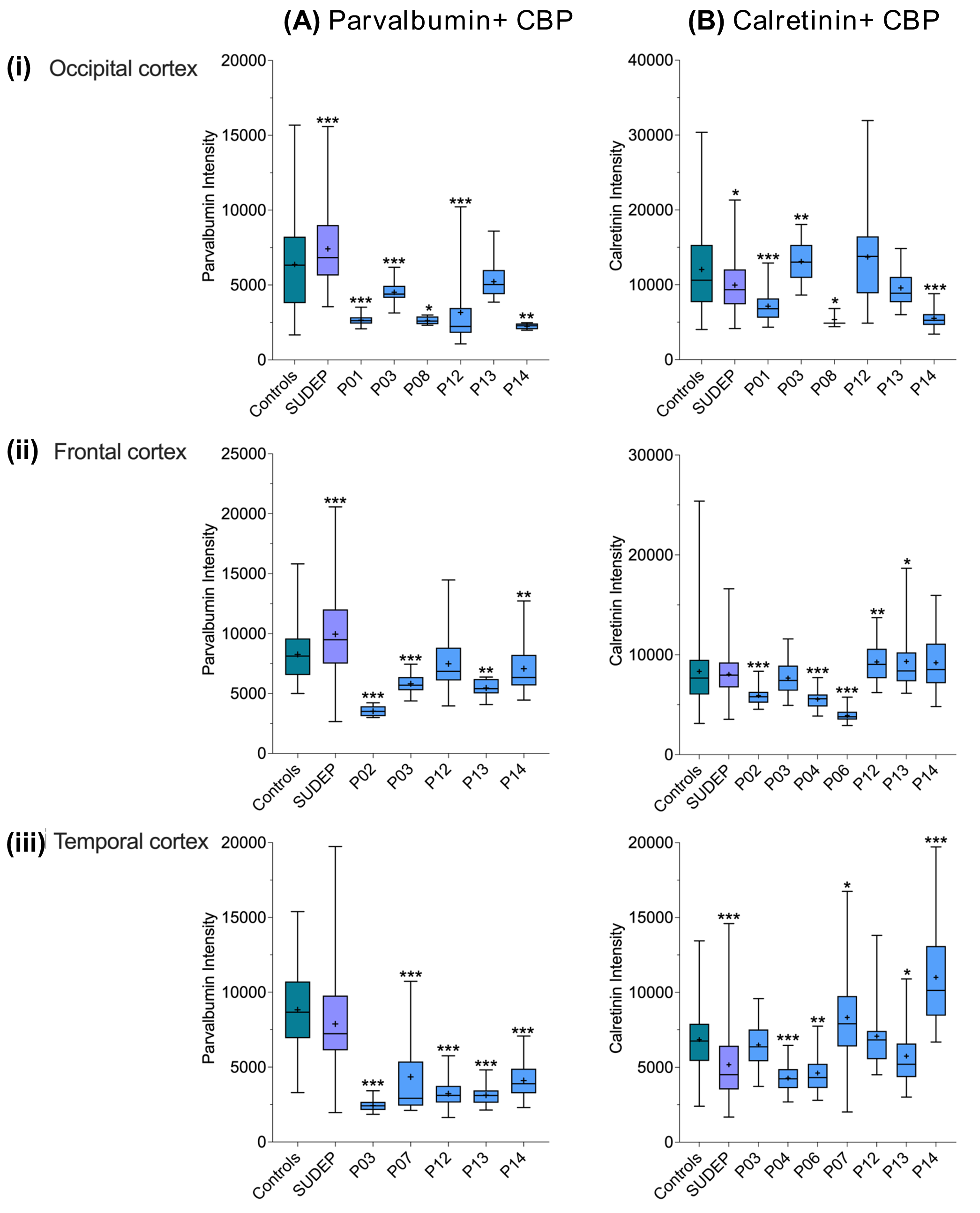

Supplement: Supplementary file 6 — Supplementary Figure S6 Calcium‐binding proteins in Alpers' syndrome. Mean optical intensity of a parvalbumin+ and b calretinin+ calcium‐binding proteins (CBP) within the (i) occipital, (ii) frontal and (iii) temporal cortices are presented as box and whisker plots; + indicates mean value. Data analysed using Kruskal‐Wallis followed by Dunn's method for multiple comparisons: *** P < 0.0001, ** P < 0.01, * P < 0.05. [file NAN-48-0-s008.tif]
