# Supplementary material for: Delineating selective vulnerability of inhibitory interneurons in Alpers' syndrome
Source: Neuropathol Appl Neurobiol. 2022 Jul 19;48(6):e12833. doi: 10.1111/nan.12833 (PMC9546160; doi:10.1111/nan.12833)
Supplement: Supplementary file 7 — Supplementary Table S1. Demographic details for patient and control tissues [file NAN-48-0-s005.docx]

**Supplementary Table 1.** Demographic details for patient and control tissues

| *Case* | *Source* | *Sex* | *Age at death* | *PMI  (hours)* | *Formalin fixation  (months / years)* | *Brain weight (grams)* | *Cause of death* | *Brain regions available* |
| --- | --- | --- | --- | --- | --- | --- | --- | --- |
| P01 | Vienna | M | 5.5m | Unknown | Unknown | Unknown | Cardiac and respiratory failure | Occipital |
| P02 | Vienna | M | 13.0m | Unknown | Unknown | Unknown | Pneumonia | Frontal |
| P03 | Newcastle Brain Tissue Resource | F | 14.0m | 12 | 1.0m | 860 | Hepatic failure | Occipital, Frontal, Temporal |
| P04 | Southampton* | M | 17.0m | Unknown | 6.0m | 380 | Unknown | Frontal, Temporal |
| P05 | NeuroBioBank | F | 18.0m | 17 | 18.8y | 580 | Respiratory failure | Occipital, Frontal, Temporal |
| P06 | Oxford* | M | 2.8y | Unknown | 4.0m | 490 | Respiratory failure | Frontal, Temporal |
| P07 | Bristol* | F | 4.0y | 24 | 3.0m | 563.1 | Respiratory failure | Temporal |
| P08 | Vienna | F | 7.0yr | Unknown | Unknown | Unknown | Pneumonia | Occipital |
| P09 | NeuroBioBank | M | 11.9y | 21 | 9.0y | Unknown | Complication of disorder | Occipital, Frontal, Temporal |
| P10 | Oxford* | M | 12.5y | Unknown | 32.0y | 1057 | Uncontrollable myoclonic epilepsy | Occipital |
| P11 | Oxford* | F | 14.0yr | 48 | 17.0y | 264 | Unknown | Occipital |
| P12 | Newcastle Brain Tissue Resource | F | 22.0y | 32 | 1.0m | 1263 | Status epilepticus | Occipital, Frontal, Temporal |
| P13 | Newcastle Brain Tissue Resource | F | 24.0y | 83 | 4.5m | Unknown | Suppurative tracheobronchitis | Occipital, Frontal, Temporal |
| P14 | Newcastle Brain Tissue Resource | F | 28.0y | 64 | 1.0m | 1352 | Status epilepticus | Occipital, Frontal, Temporal |
| S01 | Oxford* | F | 17.0m | 120 | <1.0y | 1377 | SUDEP | Occipital, Frontal |
| S02 | Oxford* | F | 4.5y | 48 | <5.0m | 1240 | SUDEP | Occipital, Frontal, Temporal |
| S03 | Oxford* | M | 10.0y | 144 | <9.0m | 1655 | SUDEP | Occipital, Frontal |
| S04 | Newcastle Brain Tissue Resource | M | 19.0y | Unknown | Unknown | Unknown | SUDEP | Occipital, Frontal, Temporal |
| S05 | Oxford* | M | 27.0y | 48 | <4.0m | 1820 | SUDEP | Occipital, Frontal |
| C01 | NeuroBioBank | F | 14.0m | 20 | 3.1y | 1000 | Coarctation of aorta | Occipital, Frontal, Temporal |
| C02 | NeuroBioBank | M | 12.7y | 15 | 9.3y | Unknown | Hanging / suicide | Occipital, Frontal |
| C03 | NeuroBioBank | M | 12.8y | 13 | 8.3y | Unknown | Drowning | Temporal |
| C04 | NeuroBioBank | F | 14.5y | 8 | 9.0y | Unknown | Streptococcal toxic shock syndrome | Occipital, Frontal, Temporal |
| C05 | Oxford* | M | 16.0y | 72 | 1.0m | 1445 | Unascertained sudden death | Occipital, Temporal |
| C06 | Edinburgh Brain and Tissue Bank | M | 16.0y | 47 | 4.0d | 1490 | Suspension by ligature | Occipital, Frontal, Temporal |
| C07 | Edinburgh Brain and Tissue Bank | F | 16.0y | 49 | 6.0d | 1720 | Sudden cardiac death | Occipital, Frontal, Temporal |
| C08 | Newcastle Brain Tissue Resource | F | 18.0y | 81 | 6.0m | 1400 | MDMA toxicity, cardiac arrest | Occipital, Frontal, Temporal |
| C09 | Edinburgh Brain and Tissue Bank | F | 24.0y | 47 | 9.0d | 1450 | Suspension by ligature | Occipital, Frontal |

**P01–P11** patients with Alpers’ syndrome, **P12–P14** adult patients with POLG-related encephalopathy, **S01–S05** sudden unexpected death in epilepsy patients (**SUDEP**; epilepsy disease control), **C01–C09** age-matched controls (no neurological or neurodegenerative diagnoses). Ethical approval for the use of post-mortem tissues were obtained: Newcastle Brain Tissue Resource (19/NE/0008); Edinburgh Brain Tissue Bank (East of Scotland Research Ethics Service REC1); NeuroBioBank; Medical University of Vienna Brain Bank, Austria; ***BRAIN UK** (19/SC/0217) Centres in Southampton, Oxford, Bristol. **F** Female, **M** Male, **PMI** Post-mortem interval, **m** months, **y** years. (removed uncommon abbreviations)
