# Supplementary material for: Delineating selective vulnerability of inhibitory interneurons in Alpers' syndrome
Source: Neuropathol Appl Neurobiol. 2022 Jul 19;48(6):e12833. doi: 10.1111/nan.12833 (PMC9546160; doi:10.1111/nan.12833)
Supplement: Supplementary file 8 — Supplementary Table S2. Primary antibodies for chromogen immunohistochemistry experiments [file NAN-48-0-s001.docx]

**Supplementary Table 2.** Primary antibodies for chromogen immunohistochemistry experiments

| *Target* | *Antibody supplier* | *Catalogue number  (RRID reference)* | *Host isotype* | *Antigen retrieval* | *Antibody dilution in TBST (4^o^C overnight)* | *Amplification and detection* |
| --- | --- | --- | --- | --- | --- | --- |
| Parvalbumin interneurons | Merck Sigma-Aldrich | P3088 (RRID:AB_477329) | Mouse IgG1 | 1mM EDTA (pH 8.0) pressure cooker (40mins) | 1:2000 | Menapath polymer kit (A. Menarini Diagnostics) and DAB chromogen  (5 mins incubation) |
| Calretinin interneurons | Swant | 6B3 (RRID:AB_10000320) | Mouse IgG1 |  | 1:2000 |  |
| Calbindin-D28K interneurons | Swant | 300 (RRID:AB_10000347) | Mouse IgG1 |  | 1:2000 |  |
| Somatostatin interneurons | ThermoFisher | PA5-82678 (RRID:AB_2789834) | Rabbit IgG |  | 1:1000 |  |
| Pyramidal neurons (neurofilament non-phosphorylated / SMI-32) | BioLegend | 801701 (RRID:AB_2564642) | Mouse IgG1 |  | 1:6000 |  |
| C-fos (marker of neuronal activation) | Abcam | Ab222699 (RRID:AB_2891049) | Rabbit |  | 1:1000 |  |
| Reactive astrocytes (glial fibrillary acidic protein) | DAKO | Z0334 (RRID:AB_10013382) | Rabbit | 0.01M Trisodium citrate (pH6.0) microwave (10mins) | 1:15,000 |  |

Endogenous peroxidase activity was quenched with a 3% hydrogen peroxide (H_2_O_2_; Sigma-Aldrich) solution for 15 minutes. Primary antibody dilution in Tris-buffered saline, 0.1% Tween 20^®^ (**TBST**, pH 7.4).
