# Supplementary material for: Delineating selective vulnerability of inhibitory interneurons in Alpers' syndrome
Source: Neuropathol Appl Neurobiol. 2022 Jul 19;48(6):e12833. doi: 10.1111/nan.12833 (PMC9546160; doi:10.1111/nan.12833)
Supplement: Supplementary file 9 — Supplementary Table S3. Antibodies for quadruple immunofluorescence assay [file NAN-48-0-s003.docx]

**Supplementary Table 3.** Antibodies for quadruple immunofluorescence assay

| ***Primary antibodies*** | | | | ***Secondary antibodies*** | |
| --- | --- | --- | --- | --- | --- |
| *Target* | *Antibody* *(dilution in TBST)* | *Host isotype* | *Company details*  *(RRID reference)* | *Alexa Fluor^TM^ secondary antibody*  *(dilution in 10% NGS/TBST)* | *Company details (RRID reference)* |
| Parvalbumin- or Calretinin-positive interneurons | Parvalbumin (1:500) | Rabbit IgG | Swant, PV27  (RRID:AB_2631173) | Goat anti-rabbit IgG 405nm  (1:100, 2 hours 4^o^C) | ThermoFisher, A31556 (RRID:AB_221605) |
|  | Calretinin (1:500) | Rabbit IgG | Swant, 7967  (RRID:AB_2619710) |  |  |
| Mitochondrial respiratory chain complex I subunit | NDUFB8 (1:100) | Mouse IgG1 | Abcam, Ab110242  (RRID:AB_10859122) | Goat anti-mouse IgG1 Biotin for amplification (1:200, 30 mins RT) | ThermoFisher, A10519 (RRID:AB_2534028) |
|  |  |  |  | Goat anti-mouse Streptavidin 546nm  (1:100, 2 hours 4^o^C) | ThermoFisher, S11225 (RRID:AB_2532130) |
| Mitochondrial respiratory chain complex IV subunit | COX1  (1:200) | Mouse IgG2a | Abcam, Ab14705  (RRID:AB_2084810) | Goat anti-mouse IgG2a 488nm  (1:00, 2 hours 4^o^C) | ThermoFisher, A21131 (RRID:AB_2535771) |
| Mitochondrial membrane | Porin / VDAC1 (1:200) | Mouse IgG2b | Abcam, Ab14734  (RRID:AB_443084) | Goat anti-mouse IgG2b 647nm  (1:100, 2 hours 4^o^C) | ThermoFisher, A21242 (RRID:AB_2535811) |

Sections were blocked in 10% normal goat serum (**NGS**) for 1 hour at room temperature. Primary antibody dilution in Tris-buffered saline, 0.1% Tween 20^®^ (**TBST**, pH 7.4). Secondary antibody dilution in 10% NGS / TBST.
